# Mesenchymal cell replacement corrects thymic hypoplasia in murine models of 22q11.2 deletion syndrome

**DOI:** 10.1172/JCI160101

**Published:** 2022-11-15

**Authors:** Pratibha Bhalla, Qiumei Du, Ashwani Kumar, Chao Xing, Angela Moses, Igor Dozmorov, Christian A. Wysocki, Ondine B. Cleaver, Timothy J. Pirolli, Mary Louise Markert, Maria Teresa de la Morena, Antonio Baldini, Nicolai S.C. van Oers

**Affiliations:** 1 Department of Immunology,; 2 Eugene McDermott Center for Human Growth and Development,; 3 Departments of Bioinformatics and; 4 Population and Data Sciences, Departments of; 5 Pediatrics,; 6 Internal Medicine,; 7 Molecular Biology, and; 8 Division of Pediatric Cardiothoracic Surgery, The University of Texas Southwestern Medical Center, Dallas, Texas, USA.; 9 Departments of Pediatrics and Immunology, Duke University Medical Center, Durham, North Carolina, USA.; 10 Division of Immunology, Department of Pediatrics, University of Washington, and Seattle Children’s Hospital, Seattle, Washington, USA.; 11 Department Molecular Medicine and Medical Biotechnology, University of Naples Federico II, Naples, Italy.; 12 Department of Microbiology, The University of Texas Southwestern Medical Center, Dallas, Texas, USA.

**Keywords:** Genetics, Immunology, Embryonic development, Genetic diseases, T cell development

## Abstract

22q11.2 deletion syndrome (22q11.2DS) is the most common human chromosomal microdeletion, causing developmentally linked congenital malformations, thymic hypoplasia, hypoparathyroidism, and/or cardiac defects. Thymic hypoplasia leads to T cell lymphopenia, which most often results in mild SCID. Despite decades of research, the molecular underpinnings leading to thymic hypoplasia in 22q11.2DS remain unknown. Comparison of embryonic thymuses from mouse models of 22q11.2DS (Tbx1^neo2/neo2^) revealed proportions of mesenchymal, epithelial, and hematopoietic cell types similar to those of control thymuses. Yet, the small thymuses were growth restricted in fetal organ cultures. Replacement of Tbx1^neo2/neo2^ thymic mesenchymal cells with normal ones restored tissue growth. Comparative single-cell RNA-Seq of embryonic thymuses uncovered 17 distinct cell subsets, with transcriptome differences predominant in the 5 mesenchymal subsets from the Tbx1^neo2/neo2^ cell line. The transcripts affected included those for extracellular matrix proteins, consistent with the increased collagen deposition we observed in the small thymuses. Attenuating collagen cross-links with minoxidil restored thymic tissue expansion for hypoplastic lobes. In colony-forming assays, the Tbx1^neo2/neo2^-derived mesenchymal cells had reduced expansion potential, in contrast to the normal growth of thymic epithelial cells. These findings suggest that mesenchymal cells were causal to the small embryonic thymuses in the 22q11.2DS mouse models, which was correctable by substitution with normal mesenchyme.

## Graphical abstract

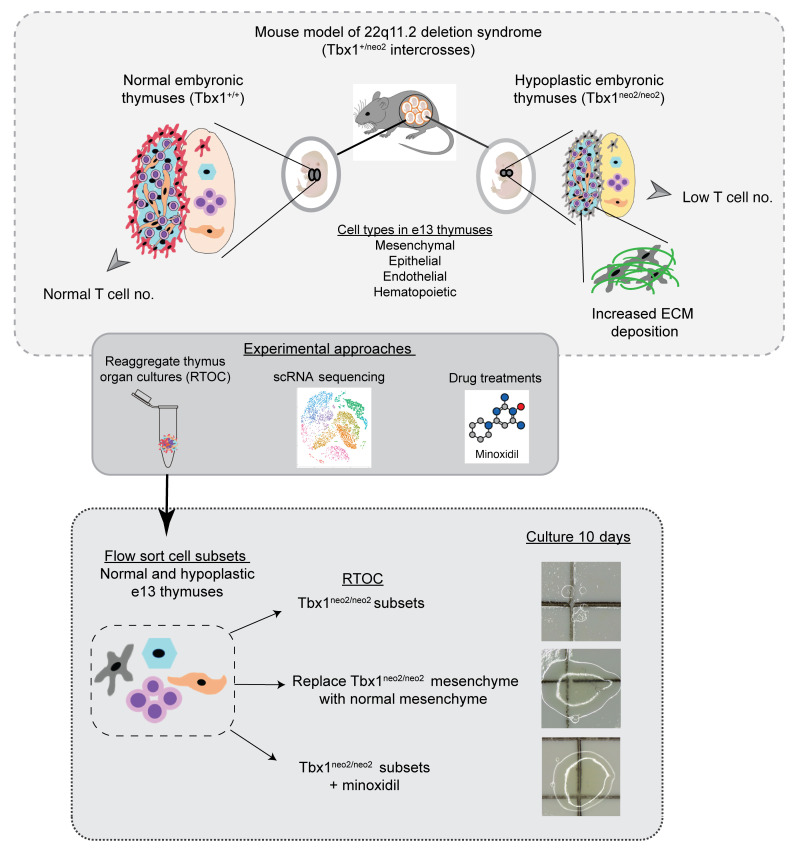

## Introduction

Chromosome 22q11.2 deletion syndrome (22q11.2DS) is the most common human microdeletion disorder reported, affecting approximately 1 of 4,000 individuals (1–4). Patients with this syndrome have developmentally linked congenital malformations, thymic hypoplasia or aplasia resulting in low T cell levels, cardiac defects, and hypoparathyroidism leading to low calcium and/or dysmorphic facial features (1, 3–5). Over time, many patients will have learning disabilities, autism, attention deficit disorders, and/or schizophrenia (1, 3, 6–9). The diverse and variably penetrant clinical presentations result from either a 3 Mb or nested 1.5 Mb deletion on chromosome 22q (4, 5). Both deletions lead to a haploinsufficiency of the T-box 1 (*TBX1*) transcription factor, a master regulator of pharyngeal patterning during embryogenesis (3, 10–13). While *TBX1* plays a key role in the congenital malformations, various other genetic and epigenetic regulators can influence the penetrance and severity of the clinical phenotypes of 22q11.2DS (4).

Sixty to seventy percent of patients with 22q11.2DS have varying degrees of T cell lymphopenia due to thymic hypoplasia (often termed DiGeorge syndrome) (1–4, 14). T cell development remains normal for most, with lower T cell numbers the prevailing clinical presentation, resulting in mild SCID (15). Less than 1% of patients with 22q11.2DS have thymic aplasia, resulting in severe immunodeficiency due to the absence of T cells (16, 17). Some T cell development can be restored for these individuals with an allogenic thymic tissue implant (18, 19). Depleted of most hematopoietic cells, the engrafted thymus is composed of stromal cells that recruit host-derived stem cells, which develop into thymocytes (18–21). This clinical approach confirms that defects in host stromal tissues (mesenchymal cells, thymic epithelial cells [TECs], and/or endothelial cells) are the basis of thymic hypoplasia in 22q11.2DS (4, 20, 22). Among these stromal cell populations, mesenchymal cells are derived from the neural crest and form the thymus capsule and vasculature (23–27). These cells interact with endothelial cells and TECs to support the formation of the thymus (25–34). TECs release chemokines to recruit thymic seeding progenitors from the bone marrow, provide growth factors for thymocyte proliferation, and express self-peptide/self-MHC complexes that dictate the selection and maturation of T cells capable of recognizing but not responding to self-peptides (32, 33, 35). TEC functionality is determined by the master transcriptional regulator Forkhead box N1 (*FOXN1*) (36–39). Autosomal recessive (AR) *FOXN1* mutations result in a nude and SCID phenotype, the latter a consequence of thymic hypoplasia and aplasia (40–45). Like 22q11.2DS, the treatment option for patients with such AR *FOXN1* mutations is an allogenic thymus implant, revealing the importance of stromal cell populations (18, 46, 47). Our understanding of the various cell types required for thymus formation and function has significantly advanced with the use of single-cell RNA-Seq (scRNA-Seq) (48). Profiling of embryonic and adult thymuses reveals many distinct mesenchymal, TEC, endothelial, and hematopoietic cell subsets in thymuses at both early developmental stages and during the aging and involution of this tissue (49–51).

Despite decades of research, the molecular defects leading to the formation of a size-restricted thymus in 22q11.2DS remain poorly defined (3, 15, 22, 52–54). Multiple mouse models of 22q11.2DS have been developed, with thymic hypoplasia most often evident in mice on a C57Bl/6 background. The small thymus severity and penetrance are *Tbx1* gene dosage dependent in mouse models, with a more penetrant hypoplasia occurring when *Tbx1* levels are less than 50% normal (10, 55–57) (Supplemental Table 1; supplemental material available online with this article; https://doi.org/10.1172/JCI160101DS1). To determine which cell populations are causal to thymic hypoplasia, we used 2 mouse models of 22q11.2DS (Df1/+ and Tbx1^neo2/neo2^) and compared the cell types necessary for the formation and function of the thymus (32). This was complemented with an analysis of human thymuses from healthy individuals and patients with 22q11.2DS. With reaggregate thymus organ culture procedures, we report that neural crest–derived mesenchymal cells were primarily responsible for the formation of size-restricted embryonic thymuses developing in the Tbx1^neo2/neo2^ mouse model of 22q11.2DS. scRNA-Seq revealed 17 distinct cell subsets in the developing fetal thymus, with the 5 mesenchymal cell subsets and 1 endothelial cell population from the hypoplastic thymuses having the most divergent transcriptomes. The differentially expressed transcripts included extracellular matrix (ECM) proteins such as collagens. We report that the drug minoxidil restored thymus size and cellularity for the hypoplastic tissues, in part by suppressing transcripts required for collagen synthesis and cross-linking.

## Results

### Hypoplastic embryonic thymuses from 22q11.2DS mouse models maintain normal thymopoiesis.

**Figure 1:**
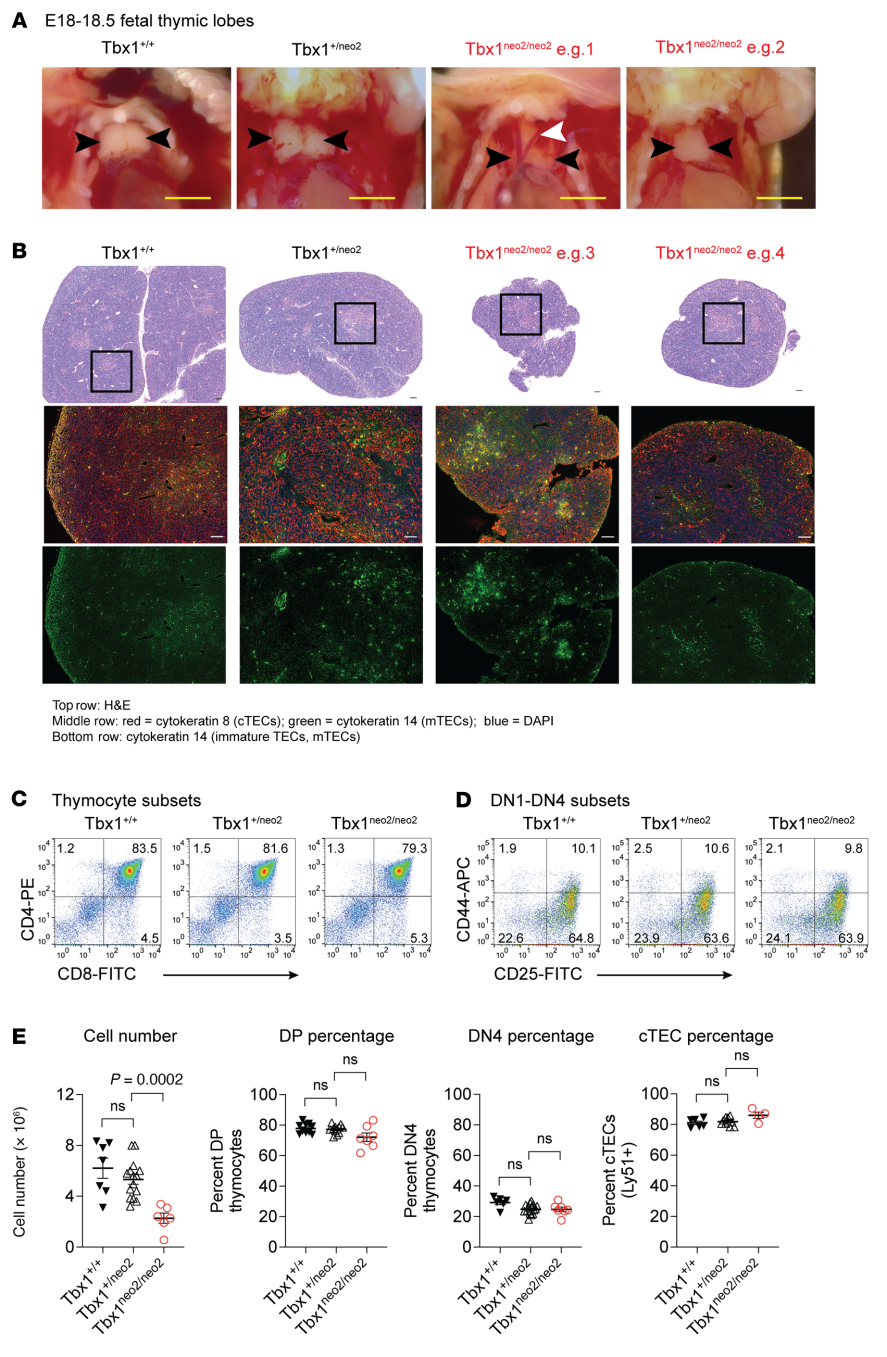
Hypoplastic embryonic thymuses isolated from 22q11.2DS mouse models have normal proportions of thymocytes and TEC subsets. (**A**–**E**) E18–18.5 embryonic thymuses were obtained from Tbx1^+/neo2^ intercrossed mouse lines. (**A**) Live cell images from the cardiothoracic regions of Tbx1^+/+^, Tbx1^+/neo2^, and Tbx1^neo2/neo2^ embryos. Thymic lobes are indicated with black arrows. An interrupted aortic arch (white arrow) often copresented with thymic hypoplasia in the Tbx1^neo2/neo2^ embryos (Tbx1^neo2/neo2^, e.g. 1). Scale bars: 50 μm. (**B**) Thymic tissue sections were processed for H&E staining and IHC. Top row: In the H&E-stained images, the cortical and medullary regions are dark and light purple, respectively. A medullary region is indicated by the boxed area. Scale bars: μm. Original magnification, ×10. Middle and bottom rows: With IHC, staining with antibodies selective for cortical (cytokeratin 8, red; middle row) and medullary (cytokeratin 14, green; bottom row) TECs is shown; DAPI staining revealed nuclei (blue; middle row). Coexpression of both cytokeratins (green and red) represents immature TECs. Original magnification, ×20 (middle and bottom rows). (**C**) T cell development was assessed by staining single-cell suspensions with antibodies selective for the CD4 and CD8 coreceptor proteins. The 4 thymocyte subsets are distinguished by electronic gating for the CD4^–^CD8^–^ (DN), CD4^+^CD8^+^ (DP), and the CD4^+^CD8^–^ and CD4^–^CD8^+^ (SP) subsets. (**D**) DN cells are further categorized by CD44 and CD25 cell-surface expression. This identifies the DN1 (CD44^+^CD25^–^), DN2 (CD44^+^CD25^+^), DN3 (CD44^–^CD25^+^), and DN4 (CD44^–^CD25^–^) subpopulations in the Tbx1^+/+^, Tbx1^+/neo2^, and Tbx1^neo2/neo2^ thymuses. (**E**) The total cell number and percentages of DP thymocytes, DN4 subpopulation of DN thymocytes, and cTECs were compared among the Tbx1^+/+^ (*n* = 7–10), Tbx1^+/neo2^ (*n* = 10–15), and Tbx1^neo2/neo2^ (*n* = 4–7) genotypes. Statistically significant differences among the 3 groups were determined by 1-way ANOVA (Brown-Forsythe and Welch tests).

22q11.2DS causes congenital malformations affecting the thymus, heart, and parathyroids (2–4). These are phenocopied in several mouse models of 22q11.2DS, including Tbx1-modified lines (10, 55–58). We noted a mildly penetrant thymic hypoplasia in the Df1/+ line, wherein a 1 Mb deletion orthologous to chromosome 22q11.2 was deleted, leading to Tbx1 haploinsufficiency (Supplemental Figure 1, A and B). The penetrance and severity of thymic hypoplasia is much higher in embryos from the Tbx1^neo2/neo2^ mouse model of 22q11.2DS due to approximately 35% normal levels of Tbx1 (Supplemental Table 1) (55). We compared thymuses between Tbx1^+/+^, Tbx1^+/neo2^, and Tbx1^neo2/neo2^ E18–E18.5 embryos derived from Tbx1^+/neo2^ intercrosses (Figure 1, A and B). We found that paired thymic lobes from the Tbx1^neo2/neo2^ embryos were consistently smaller than those of Tbx1^+/+^ and Tbx1^+/neo2^ control embryos (Figure 1A, black arrows). The magnitude of the size reduction was variable, much like that reported for individuals with 22q11.2DS (Figure 1A, e.g. 1 vs. 2, and Supplemental Table 1). In the mouse model, thymic hypoplasia copresented with an interrupted aortic arch type B, also common in patients with 22q11.2DS (Figure 1A, white arrow, and Supplemental Movie 1) (55).

The thymuses were sectioned and compared with H&E staining and IHC. Structurally, the small thymic lobes from the Tbx1^neo2/neo2^ embryos resembled those of controls (Tbx1^+/+^ and Tbx1^+/neo2^) with cortical (dark area) and medullary (lighter area) regions present (Figure 1B, see boxed areas). The magnitude of thymus size restriction was variable, with 2 representative phenotypes shown (Figure 1B, e.g. 3 and 4). Cortical TECs (cytokeratin 8), immature TECs (coexpression of cytokeratin 8 and 14), and small numbers of medullary TECs (mTECs) (cytokeratin 14) were evident, revealing a normal TEC composition in the Tbx1^neo2/neo2^ embryos (Figure 1B). Note that at embryonic stages of thymopoiesis, mTECs were limited in number, as they require single-positive (SP) thymocytes to develop. With regard to thymocyte development, the percentages of double-negative (DN) (CD4^–^CD8^–^), double-positive (DP) (CD4^+^CD8^+^), and SP (CD4^+^CD8^–^ and CD4^–^CD8^+^) stage thymocytes were similar in all the embryos (Figure 1C). Subdividing the DN thymocytes into defined developmental stages termed DN1 (CD44^+^CD25^–^), DN2 (CD44^+^CD25^+^), DN3 (CD44^–^CD25^+^), and DN4 (CD44^–^CD25^–^) revealed similar percentages of each subset in the hypoplastic lobes (Figure 1D). The major distinction with the Tbx1^neo2/neo2^ thymuses was a statistically significant reduction in cellularity in the setting of normal percentages of DP and DN thymocytes and cortical TECs (cTECs) (Figure 1E). These data concur with normal percentages of DN, DP, and SP T cells reported for most humans with 22q11.2DS (Supplemental Figure 2C) (15). Only in the rare cases of severe thymic hypoplasia and aplasia are major defects in thymopoiesis noted (Supplemental Figure 2, e.g., patient [Pt.] 2).

**Figure 2:**
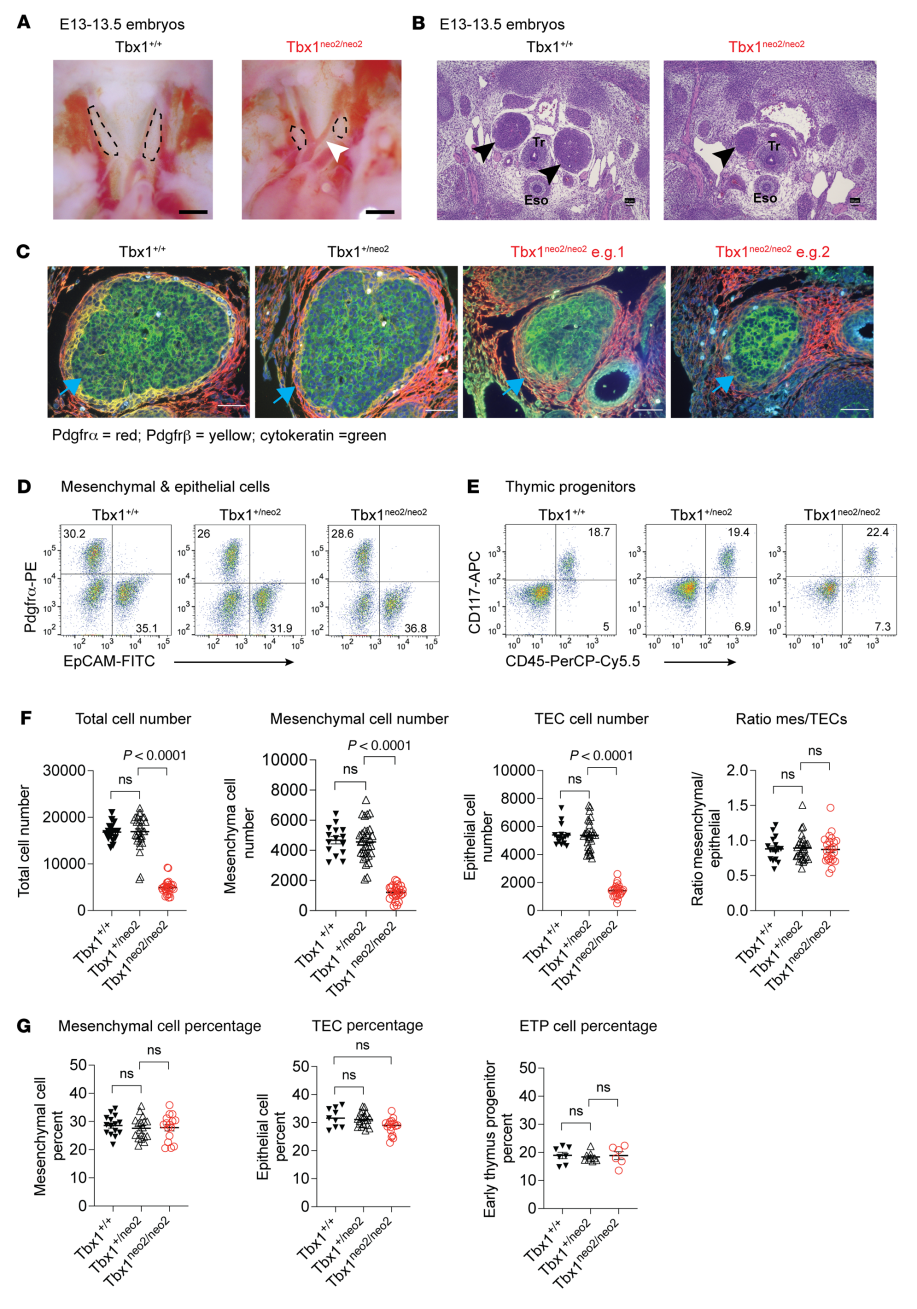
Hypoplastic embryonic thymuses have proportions of mesenchymal cells and TECs similar to those of normal control tissues. (**A**–**G**) E13–13.5 embryonic thymuses from Tbx1^+/neo2^ intercrossed time pregnant mice were genotyped and analyzed by live cell imaging, IHC, and flow cytometry. (**A**) Live cell images reveal the size and location of the developing thymus in Tbx1^+/+^, Tbx1^+/neo2^, and Tbx1^neo2/neo2^ embryos (demarcated with dotted lines). In the Tbx1^neo2/neo2^ genotype, an IAA-B was routinely visualized (white arrow). Scale bars: 0.5 mm. (**B**) Transverse sections comprising the thymus region were processed for H&E staining. Black arrows point to the thymus, with trachea (Tr) and esophagus (Eso) locations shown. Scale bars: 50 μm. In the Tbx1^neo2/neo2^ genotyped lines, the thymic lobes were not always in the same plane of the transverse section. (**C**) IHC was performed with antibodies selective for neural crest cell–derived mesenchymal cells, marking the thymus capsule (Pdgfrα, red) and thymus vasculature (Pdgfrβ, yellow), along with antibodies specific for thymic epithelial cells (EpCAM, green). Two examples of hypoplastic thymuses from Tbx1^neo2/neo2^ embryos are shown (e.g. 1 and 2). Scale bars: 50 μm. **(D** and **E)** Flow cytometric analyses of single-cell suspensions revealed the percentages of (**D**) mesenchymal (Pdgfrα^+^) and epithelial (EpCam^+^) cells and (**E**) ETPs coexpressing CD117 (c-kit) and CD45. (**F**) Enumeration of the total number of thymic cells and the specific numbers of mesenchymal and epithelial cells from multiple Tbx1^+/+^, Tbx1^+/neo2^, and Tbx1^neo2/neo2^ embryos. In addition, the ratio of mesenchymal and TECs (Mes/TECs) is shown. The Tbx1^+/+^ (*n* = 17), Tbx1^+/neo2^ (*n* = 32), and Tbx1^neo2/neo2^ (*n* = 28) genotyped embryos were used to determine cell numbers. (**G**) Percentages of mesenchymal cells, epithelial cells, and ETPs in the same thymic tissues characterized in **F**. TEC and ETP percentages were determined from a smaller number of Tbx1^+/+^ (*n* = 9–15), Tbx1^+/neo2^ (*n* = 9–20), and Tbx1^neo2/neo2^ (*n* = 6–17) mice. Statistically significant differences were established by 1-way ANOVA (Brown-Forsythe and Welch tests).

We analyzed thymuses at earlier developmental stages. Paired thymic lobes from E13–E13.5 Tbx1^neo2/neo2^ embryos were smaller and more rounded than were controls (Tbx1^+/+;+/neo2^) (Figure 2A, dotted outlines). A copresenting interrupted aortic arch type B (IAA-B) was also evident (Figure 2A, white arrow). Despite being smaller, the hypoplastic thymic lobes were structurally similar (Figure 2B, H&E staining, e.g. 1 and 2). The hypoplastic lobes were not always paired symmetrically, as seen with a missing lobe in 1 section (Figure 2B). The capsular region (Pdgfrα), vasculature (Pdgfrβ), and TECs (cytokeratin) were evident in the Tbx1^neo2/neo2^ thymuses (Figure 2C). Between E13–E13.5, mesenchymal cells and TECs were the predominant stromal cell types in the thymus, with both present at similar percentages in hypoplastic lobes as controls (Figure 2, D–G). In addition, the percentage of early thymic progenitor cells (ETPs) (CD45^+^CD117^+^CD25^–^) was equivalent (Figure 2, D and G). Overall, Tbx1^neo2/neo2^ embryonic thymuses had statistically significantly lower cell numbers, including mesenchymal cell and TEC numbers, with an average 3.4-fold lower number of cells (4,784 cells/lobe) than controls (16,526 cells/lobe) (Figure 2F). This impacted mesenchymal cells and TECs equally, as their proportions were equivalent to control proportions (Figure 2, F and G).

### Thymic lobes from 22q11.2DS embryos do not support thymic tissue expansion.

**Figure 3:**
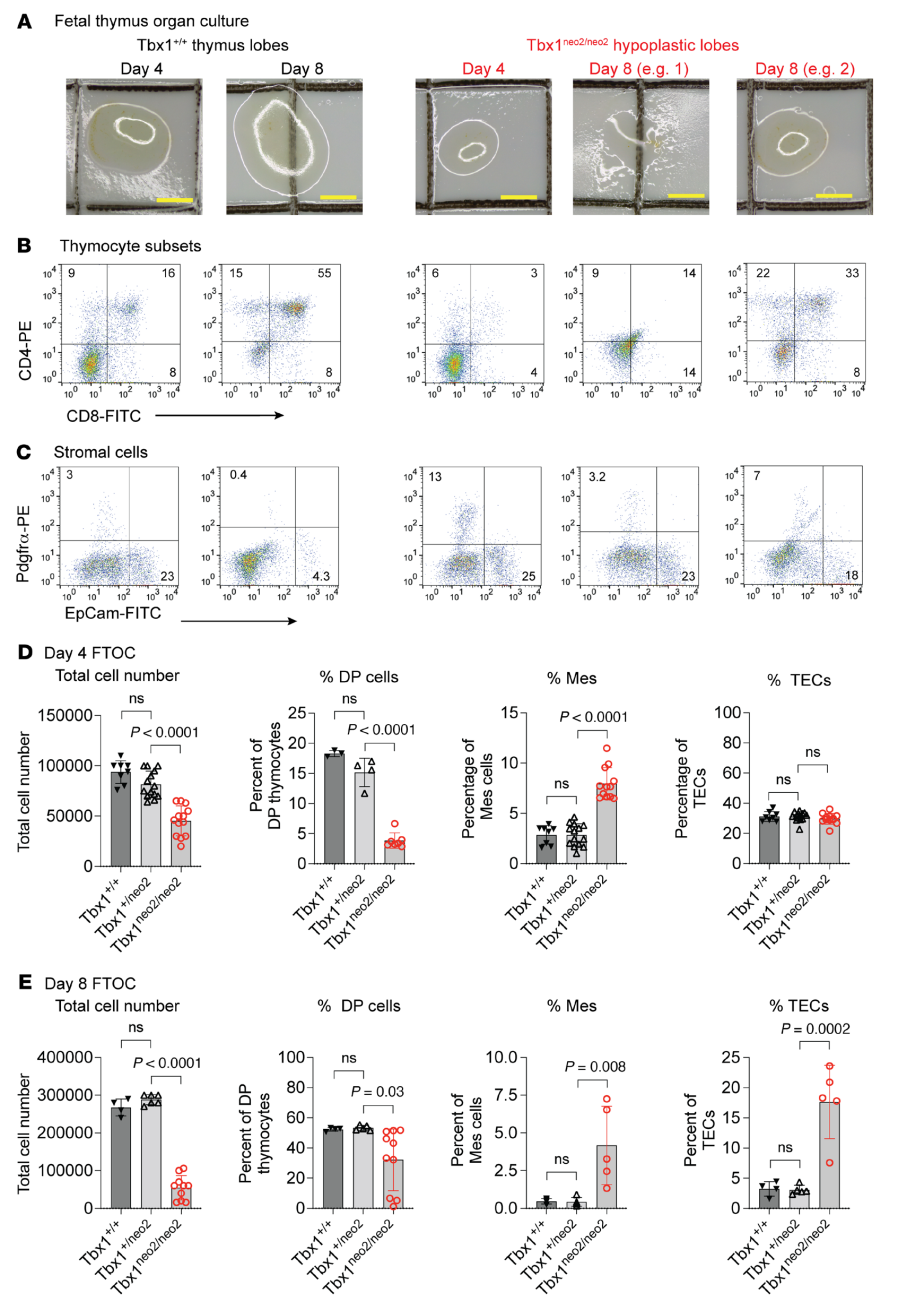
Hypoplastic fetal thymic lobes from 22q11.2DS mouse models have diminished thymopoiesis potential in culture. (**A**) Paired normal-sized (Tbx1^+/+^ or Tbx1^+/neo2^) and hypoplastic (Tbx1^neo2/neo2^) thymic lobes (E13–E13.5) were cultured for 4 days and 8 days. Live cell imaging revealed changes in thymus size, which were limited in the Tbx1^neo2/neo2^ 22q11.2DS mouse model. Scale bars: 1 mm. (**B**) T cell development was assessed by comparing the percentage of DN, DP, and SP thymocytes using electronic gating following antibody staining for surface CD4, CD8, and the TCR-β subunit expression. (**C**) The percentages of mesenchymal cells (Pdgfrα^+^) and TECs (EpCAM^+^) were determined after 4- and 8-day cultures via flow cytometric analyses. (**D**) After 4 and 8 days of FTOC, thymic lobes were processed, and total cell numbers along with the percentages of mesenchymal cells (Mes), TECs, and DP thymocytes were determined. Tbx1^+/+^ (*n* = 8), Tbx1^+/neo2^ (*n* = 14), and Tbx1^neo2/neo2^ (*n* = 12) embryonic thymuses were used. (**E**) Eight days after FTOC, the total cell numbers and percentages of live cells, DP thymocytes, and TECs were determined. Note that by day 8, relatively few Pdgfrα^+^ cells remained due to the differentiation of these cells. Tbx1^+/+^ (*n* = 4), Tbx1^+/neo2^ (*n* = 6), and Tbx1^neo2/neo2^ (*n* = 10) embryonic thymuses were used. Statistically significant differences were established by 1-way ANOVA (Brown-Forsythe and Welch tests).

To examine the growth potential of the hypoplastic lobes, we performed fetal thymus organ culture (FTOC) assays (29, 59). We found that paired thymic lobes from E13–E13.5 Tbx1^+/+^ embryos increased in size, evident on day 4 and day 8 FTOC (Figure 3, A and B). This was due to the rapid expansion of thymocytes, with 50%–60% of the cells having reached the DP stage at these time points (Figure 3B). As the thymocytes expanded and differentiated, the percentages of mesenchymal cells (Pdgfrα) and TECs (EpCAM^+^) declined (Figure 3C). Tbx1^neo2/neo2^ thymic lobes were much smaller than control lobes by days 4 and 8 of FTOC, with the tissues often dispersed on the membrane (Figure 3, A–C, e.g., 1 and 2, and Supplemental Figure 4). These thymuses had significantly lower numbers of cells and a reduced percentage of DP thymocytes, on both days 4 and 8 of FTOC (Figure 3, D and E, and Supplemental Figure 3). This correlated with the higher mesenchymal cell and TEC percentages (Figure 3, D and E). We also compared cell death and proliferation among the different genotypes. Overall, the levels of cell death, measured by coexpression of 7-AAD and annexin V, were very low (5%) in all the tissues. We noted a small decrease in the percentage of mesenchymal cell death by day 4 in the Tbx1^neo2/neo2^ FTOC (Supplemental Figure 3). Yet, thymocyte proliferation was not statistically different among the groups compared, whereas the percentage of proliferating TECs (Ki-67^+^) was slightly reduced in the Tbx1^neo2/neo2^ FTOC (Supplemental Figure 3). These findings point to a differentiation and/or functional defect among the mesenchymal cells, endothelial cells, and/or TECs. As mesenchymal cells produce growth factors to support FTOC expansion, reduced levels of these in Tbx1^neo2/neo2^ lobes could account for the reduced lobe expansion (26, 27, 29). To address this possibility, we placed paired hypoplastic lobes in FTOC surrounded by 4 pairs of control lobes. Despite the tissue expansion of the surrounding control lobes, we found that the centrally positioned Tbx1^neo2/neo2^ lobes did not expand (Supplemental Figure 4, A–D). Taken together, our results reveal that at early stages of thymus formation, the thymus from the 22q11.2DS mouse model was growth restricted in FTOC.

### Normal mesenchymal cells restore cellularity to hypoplastic thymuses.

**Figure 4:**
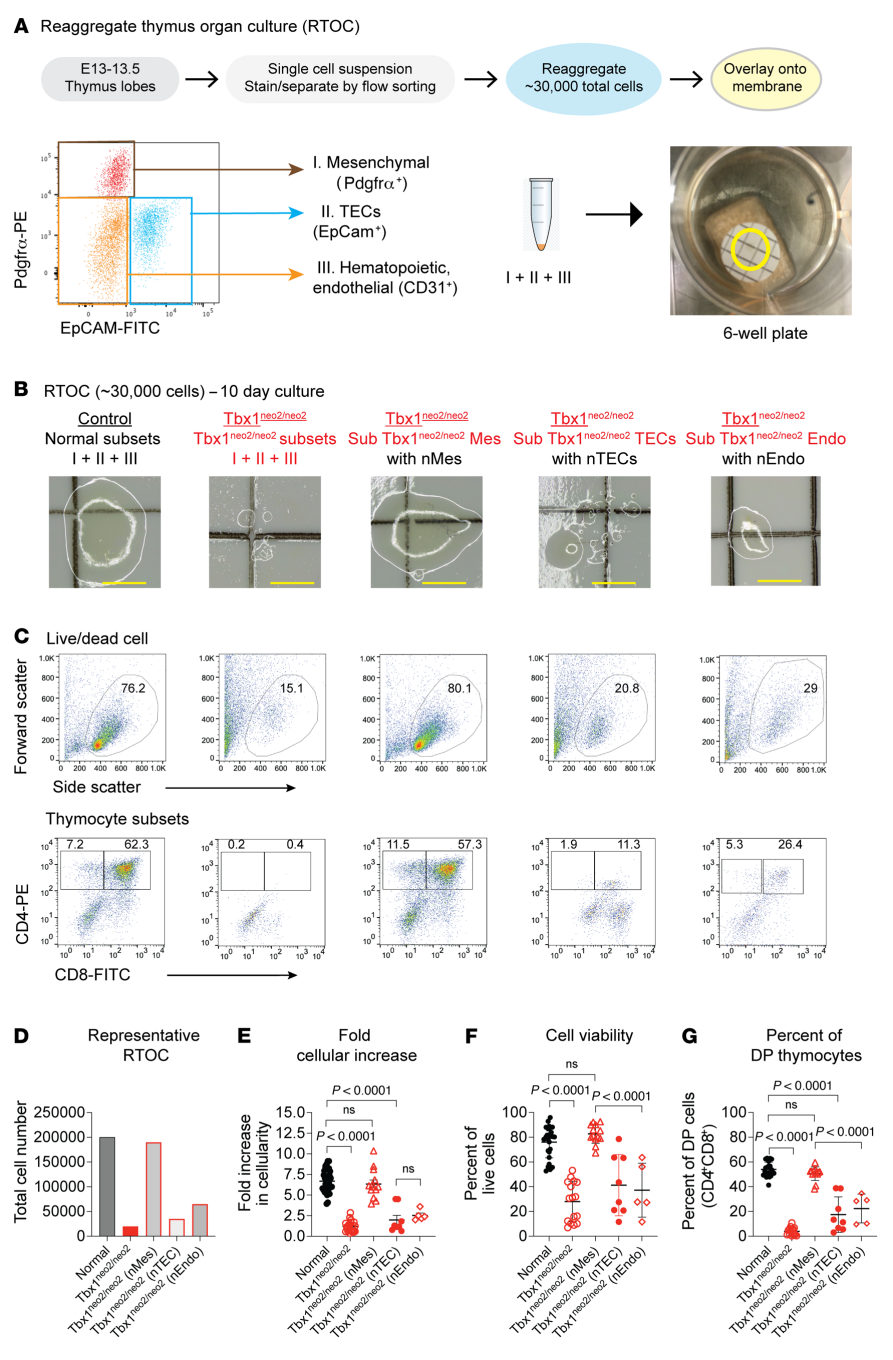
Tissue expansion is restored for hypoplastic thymuses by replacement of Tbx1^neo2/neo2^-derived mesenchymal cells with normal control cells. (**A**) Depiction of RTOC using flow-sorted cells. Single-cell suspensions from E13–E13.5 fetal thymic lobes were prepared, and mesenchymal cells (Pdgfrα^+^), TECs (EpCam^+^) and the remaining unstained cells (Pdgfrα^–^Epcam^–^, which includes ETPs, other hematopoietic cells, and endothelial cells) were sorted by flow cytometry. These 3 subgroups were reaggregated at cell ratios established with control fetal thymuses and placed onto membranes and cultured. A minimum of 30,000 cells/aggregate was needed to sustain RTOC growth with normal cells (Supplemental Figure 5). The aggregates appear as a small dot in the yellow circled area. Endothelial cell replacements required sorting of CD31^+^ cells from the remaining cell subsets prior to reaggregate culturing. (**B**) Live cell imaging was used to visualize RTOCs after 10 days of culturing. The control corresponds to the 3 subgroups of cells from Tbx1^+/+;+/neo2^ thymic lobes. In the first column, control thymuses were a combination of cells from either Tbx1^+/+^ and/or Tbx1^+/neo2^ embryos. In the second column, 22q11.2DS hypoplastic thymuses were from Tbx1^neo2/neo2^ embryos. In the third column, normal mesenchymal cells were used as substitutes for those in the 22q11.2DS tissues (Sub Tbx1^neo2/neo2^ Mes). In columns 4 and 5, normal TECs or endothelial cells were used as substitutes for Tbx1^neo2/neo2^ TECs (Sub Tbx1^neo2/neo2^ TECs) or endothelial cells (Sub Tbx1^neo2/neo2^ Endo), respectively. Scale bars: 1 mm. (**C**) Cell viability (top row) and thymopoiesis (DN to DP and then SP progression, bottom row) are shown for the cells after 10 days of RTOC. (**D**) Cumulative cell numbers are shown for a representative RTOC experiment. (**E**–**G**) The fold increase in cell numbers following 10 days of RTOC along with cell viability and the percentage of DP cells developing over this period. *n* = 37, 28, 13, 8, and 5 experiments per group, respectively, for **E**–**G**. Statistical analyses done with 1-way ANOVA (Brown-Forsythe and Welch tests).

The FTOC findings suggest that mesenchymal cells, TECs, and/or endothelial cells could be functionally compromised in 22q11.2DS (Figure 3). To define whether 1 or more of these cell types were causal to the thymic hypoplasia, we performed reaggregate thymus organ culture (RTOC) assays (Figure 4A) (59, 60). The RTOC assay was modified by initially sorting 3 different subgroups of cells by flow cytometry; mesenchymal cells (I), TECs (II), and a pool of all the remaining cell populations (III; endothelial cells and ETPs along with other hematopoietic cells). This modification enabled us to substitute different cell types from the Tbx1^+/+;+/neo2^ and Tbx1^neo2/neo2^ thymuses prior to reaggregation with an equivalent starting number of reaggregated cells (minimum of ~30,000 cells) (Figure 4A and Supplemental Figure 5).

Recombining the 3 subgroups of sorted cells from control thymuses (Tbx1^+/+;+/neo2^) resulted in thymic tissue growth (Figure 4, B–D). Thymocyte numbers increased, with DP and SP thymocyte subsets evident after 10 days of RTOC (Figure 4, B–D). Reaggregating identical numbers and proportions of the 3 cell subgroups from the Tbx1^neo2/neo2^ hypoplastic thymuses failed to sustain normal tissue growth (Figure 4, B–D). However, substitution of the Tbx1^neo2/neo2^ mesenchymal cells (Sub Tbx1^neo2/neo2^ Mes) with an equivalent number of control mesenchymal cells (Ctl Mes) restored thymic tissue expansion and thymopoiesis (Figure 4, B–D). We observed a 10-fold increase in cellularity, matching the cell numbers achieved with controls (Figure 4, D and E). Cell viability and DP cell percentages also equaled control RTOC levels (Figure 4, F and G). Substitution of TECs from the Tbx1^neo2/neo2^ thymuses (Sub Tbx1^neo2/neo2^ TECs) with normal TECs (Ctl TECs) did not sustain tissue regeneration, as the cell number, cell viability, and DP cell percentages were significantly reduced compared with control and mesenchymal cell substituted RTOCs (Figure 4, D–G). Despite representing only 1%–4% of the cells in the embryonic thymus, we also examined the contributions of endothelial cells. Substituting the Tbx1^neo2/neo2^ endothelial cells with normal ones (Sub Tbx1^neo2/neo2^ nEndo) had an effect similar to that seen with TEC-substituted cultures, with only limited tissue expansion evident (Figure 4, B–G). These experiments established that replacement of mesenchymal cells, but not TECs or endothelial cells, in the Tbx1^neo2/neo2^ thymuses restored tissue growth to normal levels. To determine whether the Tbx1^neo2/neo2^ mesenchymal cells had a negative effect on thymopoiesis, RTOCs were grown with these cells used as substitutes for normal mesenchymal cells (Sub nMes). While tissue expansion and T cell development were evident, these were not as effective as the control tissue expansion with RTOC assays (Supplemental Figure 6).

### Cellular composition of E13 embryonic thymuses.

There are multiple cell subsets in a developing fetal thymus, and how these are affected by 22q11.2DS remains undefined. To address this, we performed scRNA-Seq to identify and compare all the cell subsets present in embryonic Tbx1^+/+^ and Tbx1^neo2/neo2^ thymuses. This technique enables a precise delineation of the different cell types in a developing tissue along with key transcriptome information (51, 61, 62). Hypoplastic thymic lobes from embryos harboring mutations in the *Foxn1* transcription factor were also used in our analysis, since this gene is essential for TEC development (22, 63). AR mutations *Foxn1* (*Foxn1^1089/1089^*; c.1089_1103del15) result in thymic hypoplasia in mice that is similar to that in the Tbx1^neo2/neo2^ lines (Supplemental Figure 7).

**Figure 5:**
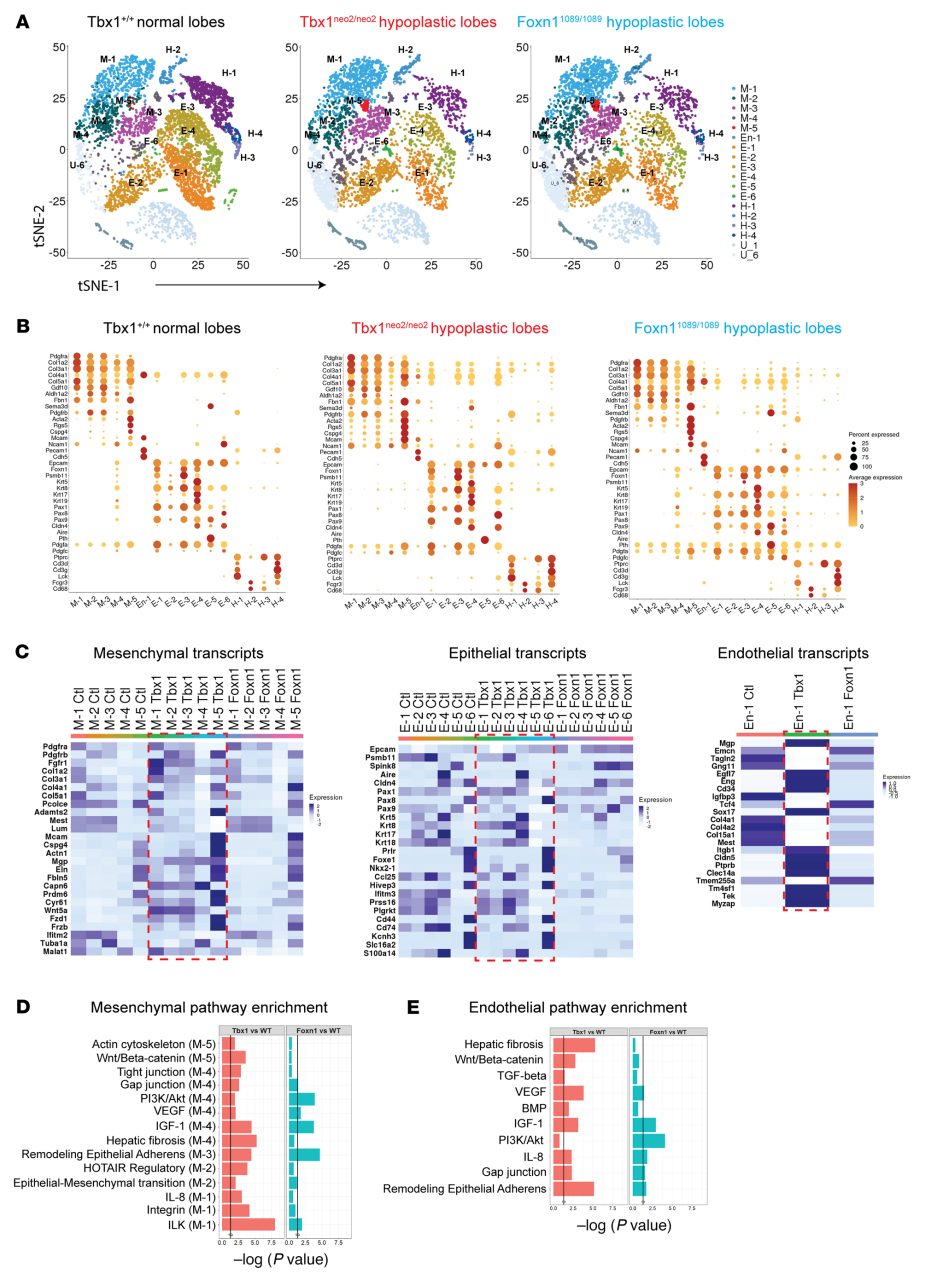
scRNA-Seq reveals distinct transcript levels in mesenchymal cells, TECs, and endothelial cells in embryonic thymuses from control, Tbx1^neo2/neo2^, and Foxn1-mutant mouse models. (**A**) Fetal thymuses, obtained from normal, Tbx1^neo2/neo2^, and Foxn1^1089/1089^ E13–E13.5 embryos, were used for scRNA-Seq. t-Distributed stochastic neighbor embedding (tSNE) plots reveal 17 distinct cell subgroups for all 3 paired thymic lobes (Tbx1^+/+^, Tbx1^neo2/neo2^, and Foxn1^1089/1089^ genotypes), with the relative percentages of these subgroups differing among the 3 genotypes. Five distinct mesenchymal cell clusters (M-1 to M-5), 6 epithelial cell groups (E-1 to E-6), an endothelial cell population (En-1), 4 hematopoietic cell types (H-1 to H-4), a RBC (U-1), and a mitochondrial signature are present in each of the thymuses. The tSNE plot for the Foxn1 hypoplastic lobes (Foxn1^1089/1089^) was generated by changing the total number cells to 6,000. (**B**) Transcripts that defined the cell subsets were compared among the 5 mesenchymal, 6 epithelial, and 4 hematopoietic cell clusters. A dot plot comparison revealed key gene expression differences among the various cell populations. (**C**) Heatmaps show the differential expression of transcripts of biological importance for mesenchymal and epithelial cell clusters along with the 1 endothelial cell cluster, respectively. Regions boxed in red represent the Tbx1^neo2/neo2^ thymus. (**D** and **E**) Pathway enrichment analyses of mesenchymal (**D**) and endothelial (**E**) clusters with DEGs revealed key distinctions between control, Tbx1^neo2/neo2^, and Foxn1^1089/1089^ fetal thymuses.

Cells from the various E13–E13.5 lobes were encapsulated in nanoliter droplets with primer-containing beads for barcoding, followed by RNA isolation, cDNA synthesis, and sequencing. We used between 5,700 and 12,440 cells per thymus, providing an average read count of 69,000/cell (Supplemental Table 2). Unsupervised hierarchical clustering revealed 17 distinct clusters in control E13.0–E13.5 thymic lobes (Figure 5A). Cellular identities were defined with singular and combinatorial gene signatures for mesenchymal cells (*Pdgfra* and *Col1A2*), epithelial cells (*EpCam* and various keratin genes), endothelial cell populations (*Cdh5* and/or *Pecam*), hematopoietic lineage cells (*Ptprc*, *Lck*, *Cd3d*, and/or *Cd3g*) and RBCs (*Hbb* genes) (Table 1). We selected additional lineage and cell type markers on the basis of their identification in previous RNA-Seq and scRNA-Seq experiments using normal embryonic thymic lobes (51, 61, 62). Of the 17 distinct cell subsets, we identified 5 mesenchymal cell subgroups (M-1 to M-5), 6 TEC subgroups (E-1 to E-6), 4 hematopoietic cell clusters (H-1 to H-4), 1 endothelial cell population (En-1), and 1 group corresponding to RBCs (U-1) (Figure 5, A and B, and Table 1). A single cluster contained mitochondrial genes (U-6). The 20 most significantly differentially expressed genes (DEGs) evident in these clusters are listed in Table 1 and Supplemental Table 3. A complete list of all DEGs among the 17 clusters is provided in Supplemental Data File 1.

The number and composition of the cell clusters in the normal and hypoplastic thymic lobes were compared first. At E13–E13.5, a normal thymus was primarily composed of nonhematopoietic cell types (Table 2). Relative to the Tbx1^+/+^ controls, the Tbx1^neo2/neo2^ thymuses lacked most of the E-5 cell population, had reduced cell numbers in E-1, E-3, and E-4 cell clusters, and more cells in E-6 and M-5 (Figure 5A and Table 2). Compared with Foxn1^1089/1089^, the Tbx1^neo2/neo2^ lobes were more affected in clusters M-5 (7-fold increase), E-5 (16-fold decrease), and E-6 (26-fold increase) (Table 2). The M-5 cell cluster expressed Pdgfrβ, which is a marker for mesenchymal cells that develop into pericytes and vascular smooth muscle (64). In E-6, the presence of prolactin (*Prl*), bone morphogenic protein 5 (*Bmp5*), and the long noncoding RNA *Rmst* (possible neural crest cell marker) suggested a mixed population in the Tbx1^neo2/neo2^ embryonic thymuses that included parathyroid cells. Both the Tbx1^neo2/neo2^ and the *Foxn1^1089/1089^* hypoplastic lobes had reduced numbers of hematopoietic cells (H-1, H-3, and H-4 clusters), consistent with the diminished effectiveness of thymopoiesis. Unique to the Foxn1^1089/1089^ thymic tissues were cellular increases in E-5 (TEC subset) and H-3 (ETPs, early thymic progenitor cells). Differences in the levels and types of transcripts were visualized with dot plots, with the percentages of cells expressing a particular transcript and the relative levels of these transcripts in 16 of the 17 cell subsets (RBC cluster excluded) shown in Figure 5B and Supplemental Figures 8, 10, and 11. We used heatmaps to reveal the transcripts associated with mesenchymal, epithelial, and endothelial cell functions (Figure 5C). As demarcated with the dashed red box, mesenchymal cell subsets of the Tbx1^neo2/neo2^ genotype had elevated expression of *Pdgfrb*, *FgfR1*, multiple collagens (*Col1a2*, *Col3a1*, *Col4a1*, *Col5a1*), and a cluster of genes coupled to ECM proteins and growth factor receptors. Among these were actinin α 1 (*Actn1*), ADAM metallopeptidase with thrombospondin type 1 (*Adamts2*), calpain 6 (*Capn6*), tropoelastin 1 (*Eln1*), elastin microfibril interfacer 1 (*Emilin1*), fibulin 5 (*Fbln5*), Forkhead box transcription factor p1 (*Foxp1*), frizzled family receptor 1 (*Fzd1*), IGF-binding protein 10 (*Igfbp10*), also known as cysteine-rich angiogenic inducer 61 (*Cyr61*)*,* matrix gla protein (*Mgp*), Pr domain–containing protein 6 (*Prdm6*, a histone-lysine methyltransferase), procollagen C-endopeptidase enhancer (*Pcolce*), and β catenin (*Ctnnb1*) (Figure 5C and Supplemental Figure 8). The 6 epithelial cell subsets (E-1 to E-6) had remarkably similar transcript levels when comparing the control and Tbx1^neo2/neo2^ thymuses (Figure 5C). Similar levels of TEC-specific transcripts were also revealed in E16.5 thymic lobes isolated from the Df1/+ mouse model of 22q11.2DS when we compared hypoplastic and normal paired lobes and controls (Supplemental Figure 1B and Supplemental Figure 9). These observations contrast with the dramatic TEC transcript differences among the E-1 to E-6 subsets that were uniquely impacted in the Foxn1^1089/1089^ thymus (Figure 5C). Therein, many of the key genes needed to support thymocyte trafficking and development were severely underexpressed (Supplemental Figure 10). Comparing the transcript levels in the single endothelial cell cluster also revealed some DEGs in Tbx1^neo2/neo2^-derived tissues that overlapped with those in the mesenchymal subsets (Figure 5C and Supplemental Figure 11). Pathway analyses revealed that Wnt/β-catenin, tight junction, hepatic fibrosis, hotair, IL-8, integrin, and ILK signaling pathways were all affected in the Tbx1^neo2/neo2^ mesenchymal subsets relative to control and Foxn1 thymuses (Figure 5D and Supplemental Table 4). Some of these same pathways were also impacted in the single endothelial cluster (Figure 5E). Taken together, the transcriptomic data provide further evidence of a mesenchymal abnormality in the Tbx1^neo2/neo2^ thymuses that affected endothelial tissues.

### Developmental alterations of the embryonic thymus in Tbx1^neo2/neo2^ lines can be overridden by blocking collagen cross-linking with minoxidil.

**Figure 6:**
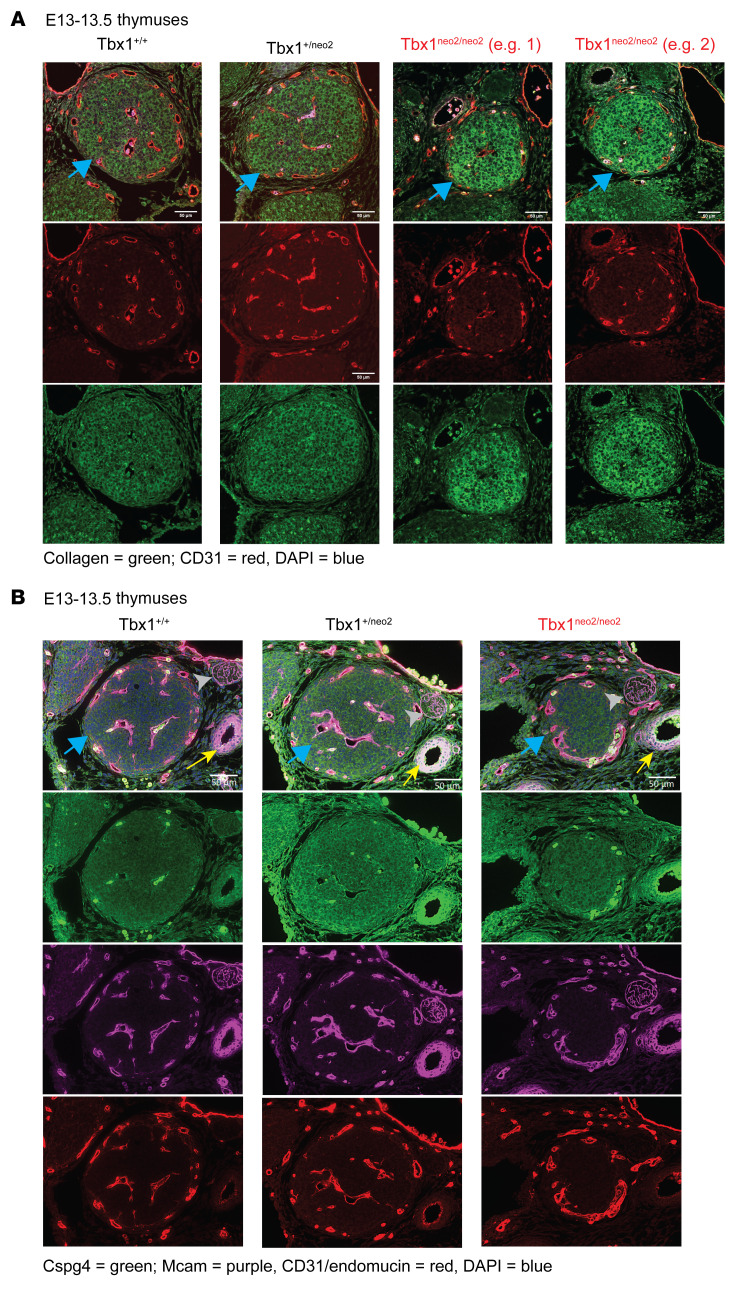
Elevated deposition of collagen is evident in hypoplastic thymuses from Tbx1^neo2/neo2^ embryos. (**A**) E13–13.5 thymuses from the indicated embryos were prepared for IHC. Staining was done with antibodies detecting collagen I (green) and a combination of CD31 and endomucin (red). Sections were prepared from Tbx1^+/+^, Tbx1^+/neo2^, and Tbx1^neo2/neo2^ embryonic thymuses. Two different Tbx1^neo2/neo2^ embryos are shown for comparative purposes. The merged image combines the collagen, CD31/endomucin, and DAPI staining (nuclei) stains. Blue arrow points to the thymus. Scale bars: 50 μm. (**B**) IHC was performed on embryos from mice of the Tbx1^+/+^, Tbx1^+/neo2^, and Tbx1^neo2/neo2^ genotypes. Antibodies selective for Cspg4 (green), Mcam (purple), and CD31/endomucin are independently shown along with a merged image comprising all the stains. The blue arrow indicates the thymus, the yellow arrow the carotid artery, and the light gray arrowhead the vagal trunk. Scale bars: 50 μm.

The scRNA comparisons revealed that mesenchymal cells had elevated transcript levels of ECM proteins such as collagen. We performed IHC staining to assess several such ECM proteins in E13.5 tissue sections (Figure 6A). Despite the reduced thymus size in the Tbx1^neo2/neo2^ embryo lines, the vasculature resembled that of the controls (Figure 6A, e.g. 1 and 2). However, the hypoplastic lobes had higher levels of collagen (Figure 6A, e.g. 1 and 2). The expression of additional ECM proteins including Cspg4 and Mcam was higher in both the Tbx1^neo2/neo2^ and Tbx1^+/neo2^ thymuses, with altered levels even more pronounced in and around the carotid artery (Figure 6B, yellow arrow). This suggests that some ECM changes were already occurring in the setting of a Tbx1 haploinsufficiency in the mouse model of 22q11.2DS. Such ECM changes were restricted to the tissues impacted by 22q11.2DS (thymus and heart), as the distribution of these proteins was normal in the vagal trunk (Figure 6B, light gray arrowhead). The endothelial layer, detected by CD31, was similar when comparing the various thymuses, suggesting that normal vascularization had occurred in the hypoplastic thymuses (Figure 6B). The scRNA-Seq data revealed an increase in the M-5 subtype, a cell population that represents pericytes, defined by Pdgfrβ expression (Figure 5C) (64). We confirmed this by flow cytometric assays. Thus, a statistically significant increase in the percentage of Pdgfrβ^+^Pdgfrα^–/lo^ cells was evident in the Tbx1^neo2/neo2^ thymuses relative to controls (Supplemental Figure 12).

**Figure 7:**
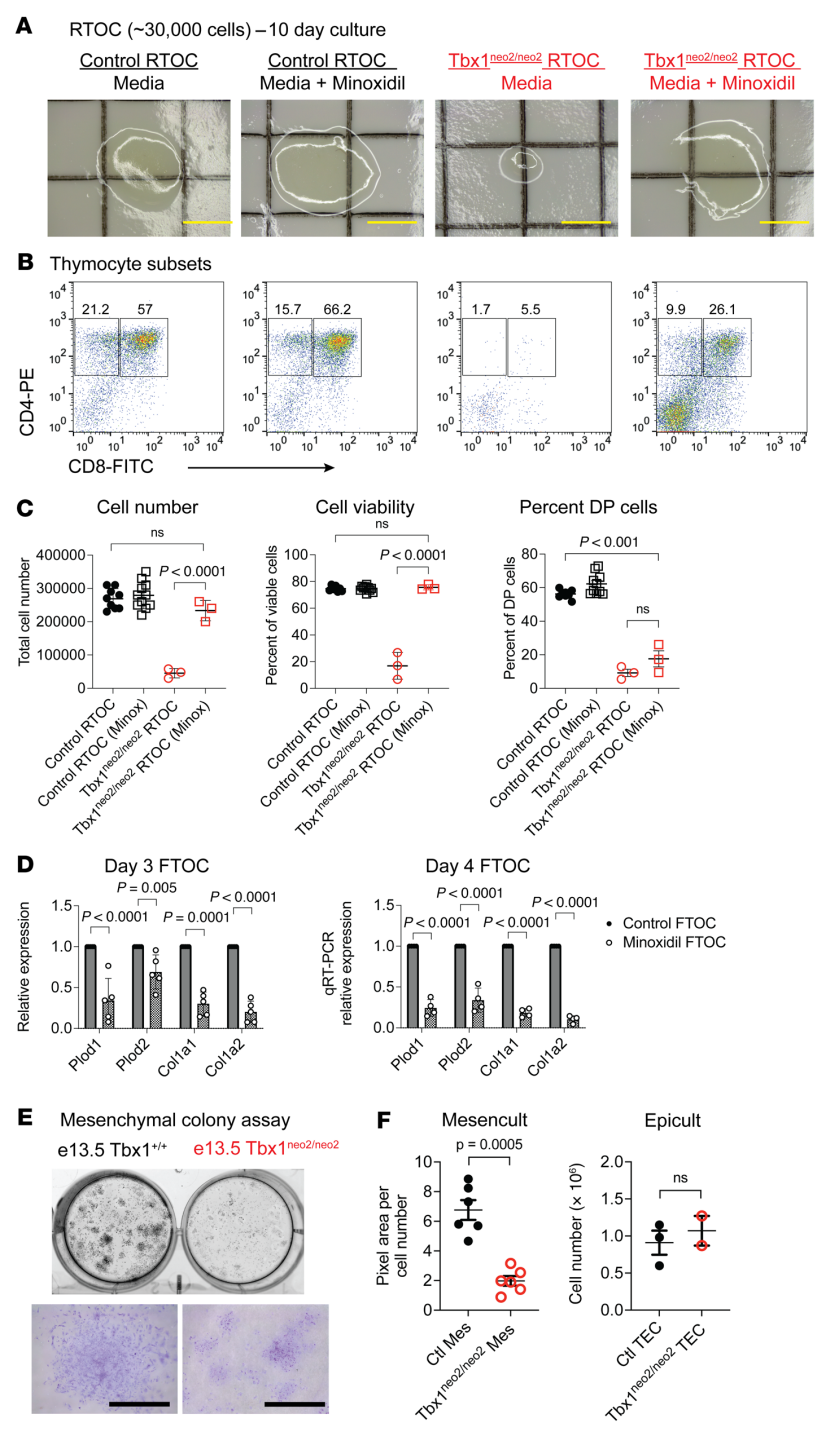
The presence of minoxidil in RTOC cultures restores tissue growth for hypoplastic thymuses. RTOC assays were performed using cell suspensions generated from E13–E13.5 fetal thymic lobes. Cells from either normal or Tbx1^neo2/neo2^ thymuses were reaggregated with equivalent starting clusters of approximately 30,000 cells/group. Cultures were maintained in media alone or supplemented with 3 μM minoxidil. (**A**) Live cell imaging revealed cell expansion after 10 days of culturing. Scale bars: 1 mm. (**B**) Thymopoiesis was compared using antibodies specific for CD4 and CD8. (**C**) Cell numbers, cell viability, and the percentage of DP cells are shown. Note that the number of cells in Tbx1^neo2/neo2^ thymuses was severely limited, as established in Figure 4, B, D, and E. *n* = 10, 10, 3, and 3 for the indicated groups, from left to right, in each panel. Statistical significance was determined by 1-way ANOVA. (**D**) Control FTOCs were grown in the absence or presence of minoxidil. On day 3 and day 4 after culturing, the cells were processed for qRT-PCR using probes detecting 2 *Plod* and 2 *Col1a* genes, along with GAPDH for normalization. Day 3, *n* = 5; day 4, *n* = 4. (**E**) Mesenchymal cells and TECs from E13–E13.5 embryonic thymuses from Tbx1^+/+^ or Tbx1^neo2/neo2^ embryos were flow sorted. Mesenchymal sorted cells were grown in MesenCult differentiation media. After 15 days of culturing, the cells were fixed, and live cell images were obtained. The well was from a 6-well tissue culture plate. Bottom image: A representative cluster of cells was imaged following crystal violet staining. Scale bars: 1 mm. (**F**) Total number of pixels in the images in **E** in conjunction with 5 additional independent experiments were calculated. These values were divided by the total number of mesenchymal cells seeded in each experiment and plotted as pixel area divided by the total cell number. This was compared with TECs grown in EpiCult. These cells were enumerated by cell counting, as shown. Statistical significance was determined by Student’s *t* test.

To determine whether increased ECM deposition and/or collagen cross-linking contributed to the thymic hypoplasia, we incubated RTOCs in the presence of several inhibitors of collagen and ECM deposition. Among these were verteporfin, minoxidil, and β-amino propionitrile (BAPN) (65–69). Verteporfin was toxic to the cultures, whereas BAPN had no effect. Control RTOCs grown with minoxidil had cellularity, cell viability, and thymocyte subset percentages similar to those without the drug (Figure 7, A–C). RTOCs with an equivalent number of cells from the Tbx1^neo2/neo2^ embryonic thymuses consistently failed to expand (Figure 7, A–C, and Figure 4). Tissue expansion in the Tbx1^neo2/neo2^ RTOCs was restored in the presence of minoxidil, as revealed by increased cellularity and improved cell viability matching those of normal controls (Figure 7, A–C). Only the percentage of DP cells, which increased in the presence of minoxidil, did not reach the same levels as those in the control RTOCs cultured with minoxidil (Figure 7C). Since minoxidil reduces the expression of enzymes linked to collagen deposition and cross-linking, including Plods and Col1a family members, we examined the transcript levels of these genes following FTOC. Normal FTOCs in the presence of minoxidil had statistically significant reduced expression of *Plod1*, *Plod2*, *Col1a1*, and *Col1a2* on both days 3 and 4 of culturing (Figure 7D). These experiments confirmed that minoxidil affected collagen deposition and cross-linking, effectively improving tissue expansion for embryonic hypoplastic thymuses from the Tbx1^neo2/neo2^ mouse model of 22q11.2DS (Figure 7D).

To expand on our observations that collagen and ECM deposition occurred in the setting of 22q11.2DS, we performed IHC comparisons with human thymic tissue sections from controls and patients. With postnatal tissues, we selectively observed increased levels of collagen in the 22q11.2DS hypoplastic human thymuses (Supplemental Figure 13). These results suggest that ECM proteins such as collagen remained elevated in thymuses from patients with 22q11.2DS after the initial formation of the thymus.

The IHC, RTOC, and scRNA-Seq results were consistent with a functional impairment among the Tbx1^neo2/neo2^ mesenchymal cells as opposed to TECs. To further address functional issues with this cell type, we performed a mesenchymal CFU assay. We flow sorted mesenchymal cells (Pdgfrα^+^) from E13–E13.5 thymuses from control (Tbx1^+/+;+/neo2^) and Tbx1^neo2/neo2^ embryos. Equivalent cell numbers were cultured in MesenCult media to induce colony formation. After 14 days in culture, the number of cell clusters (CFU/plate) was similar in control and Tbx1^neo2/neo2^ sorted mesenchymal cells, as seen with live cell imaging and crystal violet staining of one such cluster (Figure 7, D and E). However, the Tbx1^neo2/neo2^ clusters had a statistically significant reduction in mesenchymal cell expansion (Figure 7, D–F). This contrasted with the normal differentiation and expansion of TECs from control and Tbx1^neo2/neo2^ thymuses, after these cells had been sorted and cultured in EpiCult differentiation media (Figure 7F).

## Discussion

Stromal and epithelial cell defects leading to thymic hypoplasia are evident in several human conditions, including 22q11.2DS, CHARGE syndrome (featuring coloboma, heart defects, atresia choanae, growth retardation, genital abnormalities, and ear abnormalities), nude/SCID (AR *FOXN1* mutations), and diabetic embryopathies (22). While *FOXN1* mutations directly impact TECs, the cell populations affected in the other syndromes remain less well defined. We report here that thymic hypoplasia and aplasia in 22q11.2DS are linked to mesenchymal cell defects. This was confirmed by RTOC, scRNA-Seq data, and blocking of ECM deposition in developing thymuses using the Tbx1^neo2/neo2^ mouse model of 22q11.2DS. Importantly, normal fetal thymic mesenchymal cells restored thymic tissue growth and thymopoiesis when used as substitutes for cells from Tbx1^neo2/neo2^ thymuses (Figure 4).

During thymus specification and expansion, mesenchymal cells produce ECM proteins such as collagen, cell adhesion molecules, and growth factors to support both endothelial and TEC differentiation and expansion (reviewed in ref. 32). Prior studies have shown that wild-type embryonic thymuses, when stripped of the mesenchymal capsule, only expand upon readdition of this stromal tissue (29, 30). Capsule-depleted embryonic thymuses even fail to expand when transplanted under the kidney capsule, wherein adult mesenchyme surrounds the tissue (26). Despite reduced cell numbers, T cell development is normal in the capsule-stripped thymuses. In the Tbx1^neo2/neo2^ hypoplastic embryonic thymuses analyzed in the present study, higher levels of ECM proteins were apparent, and blocking collagen cross-linking with minoxidil restored tissue expansion to normal levels. Our findings are supported by recent experiments with human tissues. Blood vessel organoids, developed from induced pluripotent stem cells derived from patients with 22q11.2DS, are smaller than controls, with evident upregulation of ECM and collagen (70). This results in diminished vascular developmental processes. During embryogenesis, thymic mesenchyme differentiates into pericytes, which envelope and support the emerging endothelial vasculature (31, 71). Elimination of mesenchyme in the developing chick embryo, done by ablating the neural folds, leads to both thymic hypoplasia and cardiac outgrowth vessel defects (72). This suggests that, like the thymic hypoplasia, the congenital heart malformations in 22q11.2DS may be linked to increased collagen cross-linking and ECM deposition (13, 73, 74). This was experimentally validated with the use of minoxidil, a drug that inhibits lysyl hydroxylases (LHs, encoded by *Plods*) (65, 66, 68). Minoxidil improved hypoplastic thymus expansion in both FTOC and RTOC assays, which correlated with the inhibition of *Plod1*, *Plod2*, *Col1a1*, and *Col1a2* genes. Mesenchymal cell subsets are the predominant sources of collagens and other ECM protein in the embryonic thymus, again pointing to a key role for these cells in the phenotypes of 22q11.2DS. Minoxidil could have additional effects on thymus growth, as it reportedly increases growth factor production (75). Although our experiments did not reveal this possibility, there may be other effects imparted by minoxidil (Supplemental Figure 5). At present, our data continue to support the idea that increased collagen cross-linking and subsequent ECM deposition limit thymus expansion in mouse models of 22q11.2DS, potentially impacting mesenchymal-endothelial cell functions.

Our scRNA-Seq results provide additional evidence that mesenchymal cells and, consequently, endothelial cells were affected by 22q11.2DS in the mouse model. While both normal and hypoplastic embryonic thymuses had the same 5 mesenchymal cell subsets (Table 1: M-1 to M-5), their transcriptomes were distinct. The expression of several ECM transcripts was increased in the hypoplastic lobes. Many of these transcripts were coupled to tissue remodeling pathways including elevations in the Wnt signaling pathway (Figure 5D and Supplemental Table 4). Increased Wnt signaling disrupts thymus organogenesis (76). With regard to specific mesenchymal subsets, we found that M1-M3 were the least affected, whereas M-5 was overrepresented in the hypoplastic lobes (Tbx1^neo2/neo2^ genotype) (Table 2). M-5 marks pericytes and vascular smooth muscle cells, which are cells that surround the endothelial vasculature and regulate T cell entry and egress from the thymus (31). The M-5 population, in the Tbx1^neo2/neo2^ setting, produced more ECM and collagens (Figure 5C). With the 1 endothelial cell cluster in the Tbx1^neo2/neo2^ line, the transcriptome changes had some overlap with the mesenchymal pattern. While the endothelial cells represented only 1%–4% of all the cells in an E13 thymus, their disrupted transcriptome was likely a direct consequence of their interactions with mesenchymal cells. Current experiments are underway to define how these 2 cell types coordinate thymic tissue expansion in the embryo.

Our comparison of control, Tbx1^neo2/neo2^, and Foxn1^1089/1089^ thymuses provided strong evidence that TEC functions were normal in the 22q11.2DS mouse model. T cell development was similar, with only a delay in the developmental progression of thymocytes to the DP stage noted in the FTOC assays. scRNA-Seq revealed similar transcriptome patterns in the TEC subsets designated as E-1 to E-4 and E-6. Key transcripts needed for T cell development were present, including *Foxn1*, *Ccl25*, *Psmb11*, *Prss16*, *Cd44*, and *AIRE* (Figure 5, B and C). One difference was an underrepresentation of the E-5 population. In control thymuses, E-5 retained some parathyroid-related genes (*Pth*, *Pax8*, *Chga*), supporting prior evidence that the developing thymus contains some parathyroid precursor cells (77). We also performed quantitative reverse transcription PCR (qRT-PCR) with E16.5 thymic lobes using the Df1/+ mouse model of 22q11.2DS, comparing a hypoplastic lobe separated from its paired normal-sized lobe (Supplemental Figure 1B and Supplemental Figure 9A). The key transcripts required for TEC functions, including *Foxn1, AIRE*, and a *Foxn1* target, *E2F1*, were present at normal levels in the hypoplastic lobes (Supplemental Figure 9A). In addition, transcriptome comparisons revealed that 14 of the 22 mRNAs affected by a hemizygous deletion of the chromosome 16 genes (22q11.2 equivalent) were expressed in the developing thymus (chromosome 16 in the mouse) (Supplemental Figure 9B). A prior study compared transcripts in the third pharyngeal pouch in normal and Df1/+ embryos. *Pax1*, *Hoxa3*, *Eya1*, and *Foxn1* were found to be expressed at similar levels (78). *Pax9* and *Gcm2* were reduced (79). Taken together, the findings suggest that 22q11.2DS had a minimal impact on TEC functions despite their lower overall numbers in hypoplastic lobes. Although *Tbx1* haploinsufficiency is responsible for the heart defects in 22q11.2DS, it is not expressed in the embryonic thymus, and if forcibly expressed in this tissue, it abrogates T cell development (80).

In summary, the neural crest–derived mesenchymal cells in embryonic thymuses from mouse models of 22q11.2DS had transcriptome alterations with increased production of ECM proteins such as collagen. These changes, along with cell-cell interaction alterations, affected both mesenchymal and endothelial cells. We believe our findings are important in the context of efforts to regenerate thymuses for patients with aplasia, individuals who have thymectomies due to cardiac surgeries or the autoimmune disease myasthenia gravis, as well as for individuals undergoing rigorous chemotherapy treatments (32). Addition of appropriate mesenchymal cell populations that aid in endothelial vascularization along with TEC expansion and differentiation may provide a novel strategy for thymus organoid technologies, which are much needed in numerous clinical settings (32).

## Methods

### Human studies.

Patients in the cardiothoracic group at Children’s Health, Dallas, Texas, for corrective surgeries to treat IAA-type B, truncus arteriosus, and/or tetralogy of Fallot. Affected individuals were screened for 22q11.2DS (often clinically listed as DiGeorge syndrome). The thymus was obtained from the patients if a partial thymectomy was performed. The thymus size was variable from patient to patient. Samples were taken and processed for histological analyses. Thymic tissue sections were prepared and stained with H&E at UT Southwestern Medical Center’s Molecular Pathology Core.

### Mouse models.

Mice were housed in a specific pathogen–free (SPF) facility at UT Southwestern Medical Center. One of the murine models of 22q11.2DS used in the study, termed the Df1/+ line (Del(16Es2el-Ufd1l)217Bld), was backcrossed over 12 generations with mice on a C57BL/6 background (10, 81). These Df1/+ mice were haploinsufficient in approximately 22 orthologs of the genes spanning approximately 1 Mb on human chromosome 22q11.2 (Supplemental Figure 1A). In a second 22q11.2DS mouse model that was generated in-house, the Tbx1^+/neo2^ mouse line was used, which was already bred on a C57Bl/J background (Supplemental Figure 1A) (55, 82). There was selective targeting of *Tbx1* in this mouse line, which occurred via the insertion of neomycin into intron 5. Timed pregnancies were established by setting up breeding pairs in the late evening and screening for vaginal plugs the following morning. This was designated as day 0–0.5, primarily because the duration of the cell isolation often took an entire morning.

### RNA isolation and transcriptome analysis.

Total RNA was isolated from fetal thymic lobes, the pharyngeal region, or sorted cells. RNA was prepared and purified using the miRNana kit (Ambion, Thermo Fisher Scientific), and for small numbers of cells, the MicroRNA Isolation kit (Zymo Research) was used. Contaminating DNA was removed by DNase treatment (Applied Biosystems, Thermo Fisher Scientific) or in-column DNase digestion (Zymo Research). RNA was reverse transcribed (Applied Biosystems, Thermo Fisher Scientific) to cDNA. qRT-PCR was performed with SYBR qPCR Master Mix (Thermo Fisher Scientific) to check the expression of *Plods* and ECM genes, which were normalized to *Gapdh*. scRNA-Seq and data analysis are described in the Supplemental Methods.

### FTOC and RTOC.

FTOC assays and antibodies used for staining are detailed in the Supplemental Methods. For cell viability and proliferation assays, FTOC was performed for 4 and 8 days. After 4 days in FTOC, a single-cell suspension of lobes was made and divided into 2, and 1 set was stained with anti–EpCAM-FITC, anti–PDGFRα-PE, anti–CD45-APC Cy7, and anti–annexin V antibodies. Annexin V staining was performed at the last step using an annexin staining buffer (0.01 M HEPES, 140 mM NaCl, and 2.5 mM CaCl_2_). 7-AAD was added 10 minutes prior to analysis. The second set of cells was stained with anti–EpCAM-FITC, anti–PDGFRα-PE, and anti–CD45-APC-Cy7 antibodies, followed by intracellular staining with anti–Ki67-APC antibody following the manufacturer’s recommendations (Invitrogen Fix and Perm kit, Thermo Fisher Scientific). Analyses were done on a BD FACSAria Zelda.

For reaggregate assays, normal and hypoplastic fetal thymic lobes, at a gestational age between E13.0 andE13.5, were isolated and collected in thymus organ culture (TOC) media. The media consisted of RPMI, 20% FCS supplemented with HEPES, l-glutamine, sodium pyruvate, penicillin, streptomycin, 5 × 10^–5^ M 2-mercaptoethanol, and nonessential amino acids. A minimum of 6–8 hypoplastic lobes were needed for a single RTOC assay (30,000 cells total). Lobes were washed with PBS and digested in 0.25% trypsin and 0.02% EDTA at 37°C for 6–10 minutes, followed by pipetting until single-cell suspensions were obtained. Digestion was stopped by addition of TOC media. Cells were washed, resuspended in volumes of less than 250 μL/6–20 lobes, and an aliquot counted with a hematocytometer. Cells were stained with antibodies specific for mesenchymal cells (Pdgfrα-PE) and TECs (EpCAM-FITC) under sterile conditions. After washing, the cells were sorted into 3 populations: mesenchymal, epithelial, and the remaining cells (EpCAM^–^Pdgfrα^–^; precursor thymocytes, DCs, endothelial cells, macrophages). Sorting was done with an FACSAria Zelda machine. RTOC was performed by reaggregating the 3 cell populations — EpCAM^+^ (~30%), Pdgfrα^+^ (~30%), and EpCAM^–^Pdgfrα^–^ (~40%) — in a 1.5 mL tube in varying combinations (Figure 4A). Cells were centrifuged consecutively for 5 minutes and 10 minutes at 100*g* and 400*g*, respectively. After the second spin, the supernatant was removed, leaving behind 2–4 μL aggregated cells, which were placed on ice for 10 minutes. The cell pellet was gently dispersed, and the mixture was drawn into a pulled glass pipette and delivered as a single drop onto a Millipore nitrocellulose filter (MilliporeSigma). The filter was placed on top of a sterilized foam sponge (2 mm thick) in a single well of a 6-well tissue culture plate (60 mm diameter). The foam sponge had been soaked in 3 mL TOC medium, with air pockets removed by gentle compression with the flat end of a 1 ml syringe plunger. Reaggregated thymic lobes were cultured in a CO_2_ incubator for 10 days at 37^o^C with an input of 7.5% CO_2_. After 10 days of organ culture, reaggregated thymic lobes were harvested in PBS (Ca^2+^- and Mg^2+^-free) supplemented with 2% FCS, and single cells were prepared by gentle squishing and pipetting of the lobes. Cells were counted using a hematocytometer. An aliquot was used for flow cytometric analysis after staining the cells with antibodies against CD8-FITC, CD4-PE, and TCRβ-PerCP-Cy5.5. Samples were analyzed on a FACSCaliber (BD Biosciences), and data were analyzed with FlowJo software (Tree Star). For RTOC assays in the presence of minoxidil, single-cell suspensions from the thymic lobes were prepared. The cells were reaggregated in batches of 30,000 cells/group for RTOC. As indicated, the media were supplemented with 3 μM minoxidil in certain cultures. RTOCs were performed for 10 days at 37°C with an input of 7.5% CO_2_. Minoxidil-supplemented media were renewed every 4 days. After 10 days, the lobes were processed into a single-cell mixture. Cells were stained with antibodies specific for various cell-surface proteins.

### IHC analyses.

E10.5–E13.5 embryos and E18.5 fetal thymuses and fragments from human thymuses were fixed for 24 hours in 4% paraformaldehyde (in PBS) at 4°C. These were then dehydrated in a stepwise ethanol gradient of 25%, 50%, 75%, and 100% ethanol, prepared in PBS. After a subsequent wash in xylene, the tissues were embedded in paraffin and sectioned (4 μm thick). Slides were deparaffinized in xylene and rehydrated using a descending ethanol gradient (100%, 95%, 90%, 80%, 70%, and 50% ethanol). Antigen retrieval was performed for 15 minutes at 95°C in Antigen Retrieval R Buffer A, pH 6 (Electron Microscopy). Slides were blocked in CAS Block (Invitrogen, Thermo Fisher Scientific) for 2 hours at room temperature. Anti–cytokeratin 14, anti-cytokeratin, anti-Pdgfrα, anti-Pdgfrβ, anti–E cadherin, anti-laminin, anti-CD31, anti-endomucin, anti–collagen I, anti-Mcam, anti-Cspg4, and anti-SMA antibodies were used to stain slides overnight at 4°C. The antibodies are listed in Supplemental Table 5. Secondary antibodies were used according to the manufacturer’s instructions (Invitrogen, Thermo Fisher Scientific). The slides were stained with DAPI (Molecular Probes) prior to being mounted with Prolong Gold Anti-fade Reagent (Invitrogen, Thermo Fisher Scientific). Images were taken on a Keyence Fluorescence microscope, and images were analyzed using ImageJ software (NIH). H&E staining was performed as described previously (83). Images were also taken on a Leica TCS SP5 confocal microscope and analyzed using ImageJ. Sections stained with H&E were imaged on an Axiovert 200M inverted fluorescence microscope (Zeiss).

### Mesenchymal and epithelial cell differentiation assays.

E13–E13.5 control and Tbx1^neo2/neo2^ thymic lobes were prepared as per the RTOC experiments. Mesenchymal cells (Pdgfrα^+^) and TECs (EpCam^+^) were isolated by flow sorting as in the RTOC assays. Between 6,000 and 8,000 cells/experiment were seeded onto 6-well tissue culture plates containing RPMI media supplemented with MesenCult Expansion or EpiCult Expansion media for murine cells (both from STEMCELL Technologies). The cells were cultured at 37°C in 7.5% CO_2_. The culture medium was changed every 4 days. After 14 days, the cell colonies in the mesenchymal cultures were rinsed in PBS and then fixed in 100% methanol. Clusters were visualized by staining in 1% Crystal Violet Solution (Sigma Chemical). The plates were photographed using a ChemiCoc Imaging system (Bio-Rad). Adherent colonies containing more than 20 cells were counted as a colony. For epithelial cells, the colonies were dispersed with 0.25% trypsin, washed, and enumerated with a hemocytometer.

### Data and materials availability.

The Df1/+ and Tbx1^+/neo2^ mouse lines described in this study were obtained from outside investigators, as indicated in this article. The scRNA-Seq data were deposited in the NCBI’s Gene Expression Omnibus (GEO) database (GEO GSE170686).

### Statistics.

Statistically significant differences among the different test groups were determined by 1-way ANOVA. A *P* value of less than 0.05 was considered significant. Data represent the mean ± SEM. As indicated in the figure legends, for 1-way ANOVA, Brown-Forsythe and Welch tests were sometimes applied, as indicated. A 1-tailed Student’s *t* test was applied for certain experiments in which only 2 distinct samples were compared, as indicated in the figure legends.

### Study approval.

Informed consent was obtained for human studies under a protocol approved by the IRB of UT Southwestern Medical Center (STU-072010-003, STU-2019-1087). The animal work described in this study was approved and conducted under the oversight of the UT Southwestern Institutional Animal Care and Use Committee (APN numbers 2015-101163 and 2015-101247).

## Author contributions

PB, QD, MTDLM, and NSCVO conceptualized the study. PB, QD, AK, CX, ID, AM, CAW, OBC, AB, and NSCVO designed the study methodology. PB, QD, AK, CX, ID, AM, CAW, OBC, TJP, MLM, MDLM, AB, NSCVO performed studies. PB, QD, AK, MDLM, AB, and NSCVO designed experiments and interpreted results. CAW, MDLM, and NSCVO acquired funding. CAW, TJP, MLM, MTDLM, and NSCVO conducted clinical discussions. NSCVO supervised the study. QD and NSCVO wrote the original draft of the manuscript. PB, QD, CAW, TJP, MLM, MTDLM, AB, and NSCVO reviewed and edited the manuscript.

## Supplementary Material

Supplemental data

Supplemental data set 1

Supplemental video 1

## Figures and Tables

**Table 1:**
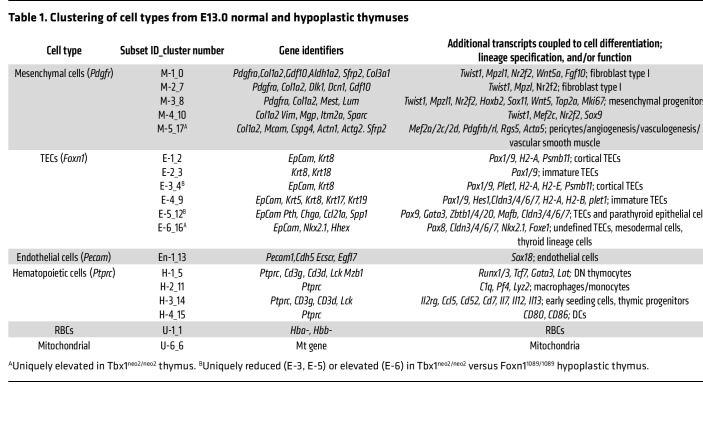
Clustering of cell types from E13.0 normal and hypoplastic thymuses

**Table 2:**
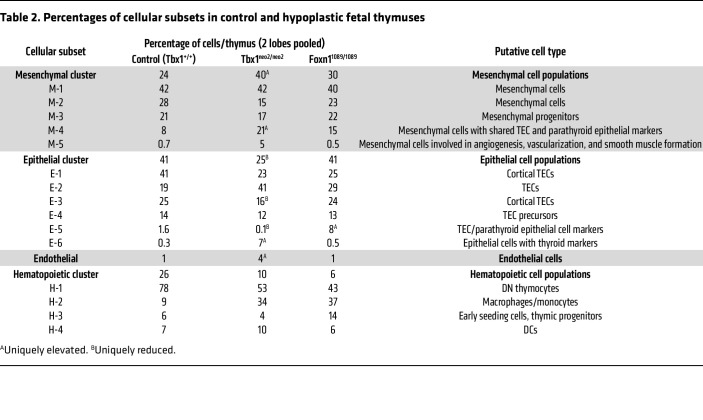
Percentages of cellular subsets in control and hypoplastic fetal thymuses

## Version 1 09/22/2022

In-Press Preview

## Version 2 11/15/2022

Electronic publication
