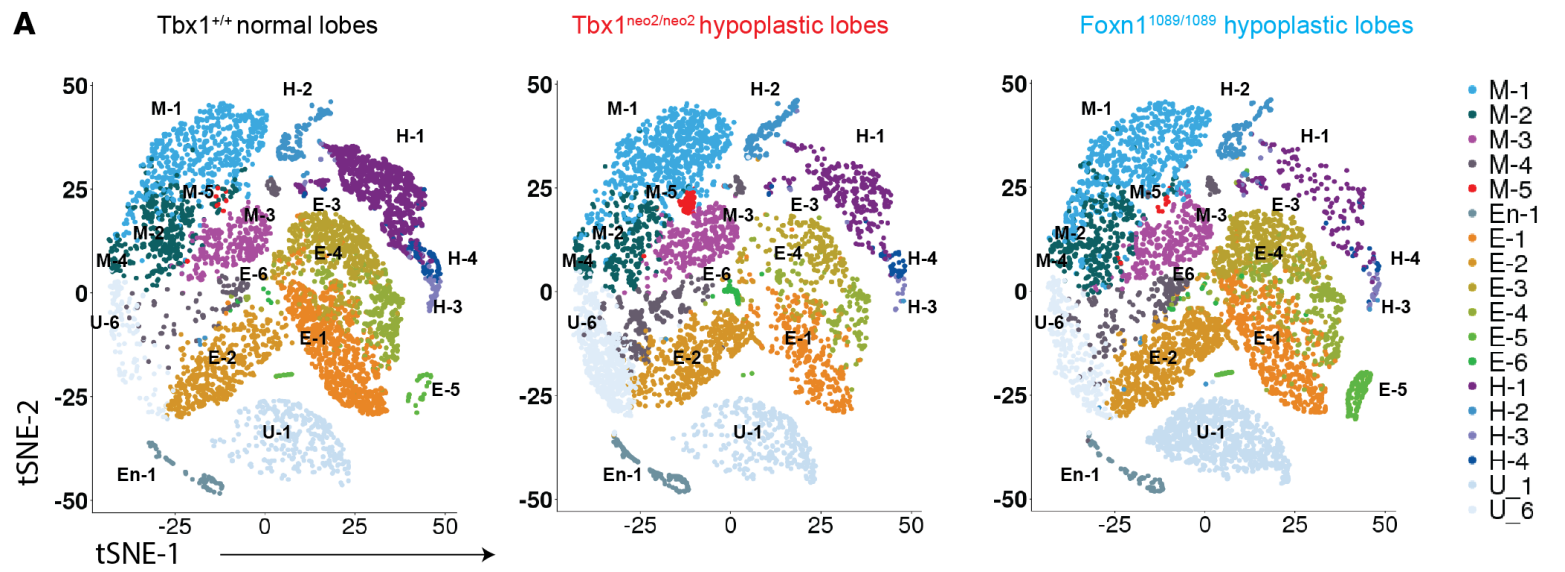# Mesenchymal cell replacement corrects thymic hypoplasia in murine models of 22q11.2 deletion syndrome

**DOI:** 10.1172/JCI173145

**Published:** 2023-07-03

**Authors:** Pratibha Bhalla, Qiumei Du, Ashwani Kumar, Chao Xing, Angela Moses, Igor Dozmorov, Christian A. Wysocki, Ondine B. Cleaver, Timothy J. Pirolli, Mary Louise Markert, Maria Teresa de la Morena, Antonio Baldini, Nicolai S.C. van Oers

Original citation: *J Clin Invest*. 2022;132(22):e160101. https://doi.org/10.1172/JCI160101

Citation for this corrigendum: *J Clin Invest*. 2023;133(13):e173145. https://doi.org/10.1172/JCI173145

In the original version of Figure 5A, the images for Tbx1^neo2/neo2^ and Foxn1^1089/1089^ hypoplastic lobes were duplicates. The correct figure part is below. The HTML and PDF files have been updated online.

The authors regret the error.

## Figures and Tables

**Figure:**