# Supplementary material for: Mesenchymal cell replacement corrects thymic hypoplasia in murine models of 22q11.2 deletion syndrome
Source: J Clin Invest. 2022 Nov 15;132(22):e160101. doi: 10.1172/JCI160101 (PMC9663160; doi:10.1172/JCI160101)
Supplement: Supplemental data [file jci-132-160101-s040.pdf]

Supplementary Materials

Methods

Flow cytometry and Fetal Thymic Organ Culture. Fetal thymic lobes were isolated between e13-13.5 gestational age under a dissecting microscope using fine forceps. Paired thymic lobes were placed atop Millipore nitrocellulose filters (0.45 μm thick, 13 mm diameter) resting on a sterilized foam sponge (2 mm thick) in a 6-well tissue culture plate (60 mm diameter). The foam sponge is soaked in 2 ml of thymic organ culture (TOC) media (RPMI-1640 media supplemented with 20% FCS, 100 U/ml penicillin, 100 ug/ml streptomycin, 50 μM 2-mercaptoethanol, 4 mM L-Glutamine, 10 mM HEPES, 1 mM nonessential amino acids, and 1 mM sodium pyruvate). FTOC was continued for 4 and 8 days at 37°C with 7.5% CO_2 (1). After 4- and 8-days organ culture, thymic lobes were harvested individually in PBS (Ca^{2+} and Mg^{2+} free) supplemented with 2% FCS, dispersed by gently squishing/pipetting to make single cell suspensions. Cells were counted using a hematocytometer and stained for flow cytometry analysis with the following antibodies: CD8-FITC, CD4-PE and TCRβ-PerCP-Cy5.5 and CD69-APC. For DN profiling, single cell suspensions were stained with CD44-APC and CD25-FITC along with CD8-PE, CD4-PE, B220-PE, NK1.1-PE, gd TCR-PE, CD11b-PE, CD11c-PE, CD19-PE, Ter-119-PE and CD45-PerCP-Cy5.5. The PE channel was used as a dump channel to enable a characterization of true DN thymocytes. Samples were analyzed on a FACS Caliber (BD Bioscience), and data was analyzed via FlowJo (Tree Star Inc.)

Single cell RNA sequencing information and data analysis. Cell Ranger 3.0.0 (10X Genomics) was used to process the raw sequencing data. BCL files were converted to FASTQ files and aligned to mouse (mm10) reference genome. Transcript counts of each cell were quantified using UMI and valid cell barcode. The gene expression matrix from cell ranger was used as input to Seurat R package (v3.0.0) for the downstream analysis (2). Cells with less than 200 genes per cell and very high mitochondrial gene content were filtered out. Global-scaling normalization method “Log Normalize” was used for normalization. Subset of genes exhibiting high variation across the single cells was determined. The highly variable genes were calculated using “FindVariableFeatures” module in Seurat. Here average expression and dispersion per gene is calculated and features are divided into bins to get z-scores for dispersion per bin. “FindIntegrationAnchors” and “Integrate module” in Seurat was used to find anchors and “integrate 3” Seurat objects corresponding to normal fetal thymus ($Tbx1^{+/+}$) 22q hypoplastic lobes ($Tbx1^{neo2/neo2}$) and Foxn1 hypoplastic lobes (Foxn11089/1089). Seurat integrated analysis was performed across samples at different biological conditions. Data was then scaled, and dimensional reduction was performed with principal component analysis. Seurat integrated analysis was performed across samples at different biological conditions. For the sample, a Shared Nearest Neighbor (SNN) Graph was constructed with “FindNeighbors” module in Seurat by determining the k-nearest neighbors of each cell. The clusters were then identified by optimizing SNN modularity using the “FindClusters” module. This allowed for a sensitive detection of rare cell types. We obtained 18 clusters with a resolution of 0.3. TSNE plot was generated using the DimPlot module in Seurat. Each cluster was compared to all other clusters using Wilcoxon Rank Sum test to test for significant differentially expressed genes. The genes identified as relatively overexpressed in a cluster as compared to all other cells were termed as “markers”. Clusters were named based on gene markers specific to various cell types.

Differential gene expression testing was performed using “FindMarkers” module in Seurat between normal fetal thymus ($Tbx1^{+/+}$), 22q.11.2DS hypoplastic lobes ($Tbx1^{neo2/neo2}$) and

Foxn1 hypoplastic lobes (Foxn11089/1089) for all cell types. To observe the changes in gene expression values, VlnPlot module of Seurat was used to generate violin plots for top differentially expressed genes in mesenchymal and epithelial cell types. Pathway analysis of the top differentially expressed genes with p-value < 0.01 was performed using Ingenuity pathway analysis (IPA) software (QIAGEN, Redwood City, www.qiagen.com/ingenuity and https://www.qiagenbioinformatics.com/products/ingenuity-pathway-analysis/). Trajectory analysis was performed using monocle2 (3). Monocle defines pseudo time as the progress of cell with respect to changes in cell's transcriptional state. We used Monocle single cell trajectory analysis algorithm to learn the trajectory of cells. Briefly, differentially expressed genes between mesenchymal subtypes in normal fetal thymus, ($Tbx1^{+/+}$), 22q11.2DS hypoplastic lobes ($Tbx1^{neo2/neo2}$) and Foxn1 hypoplastic lobes ($Foxn1^{1089/1089}$) were selected and reverse graph embedding algorithm was used to reduce the dimension of data. Next the cells were ordered to learn the trajectory describing changes in the transcriptional state from root node to branch leaves. Genes changing as function of pseudo time were plotted using module plot_genes_in_pseudotime. Genes showing similar trends in pseudo time were clustered using plot_pseudotime-heatmap module. The scRNA-seq dataset has been deposited in the Gene Expression Omnibus database (GEO accession 170686)

Reagents and supplies are listed in Table S5

Supplemental Figure Legends

Supplemental Fig. 1. **Embryos from the Df1/+ mouse model of 22q11.2DS exhibit an infrequent hypoplasia of the thymus.** (**A**) The Df1/+ mouse line lacks a 1 Mb deletion on murine chromosome 16, orthologous to human 22q11.2. Several of the key genes haploinsufficient in this line responsible for the diverse phenotypes of 22q11.2DS include *Tbx1*, *Dgcr8*, and miR-185, as indicated in the map of this chromosomal region. This contrasts the $Tbx1^{+/neo2}$ line, wherein a neomycin was inserted in the opposite orientation within intron 5 of *Tbx1*, resulting in a selective targeting of Tbx1. (**B**) Live cell imaging was used on the cardiothoracic regions of an e16.5 Df1/+ embryo to reveal the thymic lobes. A hypoplastic lobe from the right side of the mouse and an adjoining normal sized lobe were found in several embryos, consistent with prior reports.

Suppl. Fig. 1

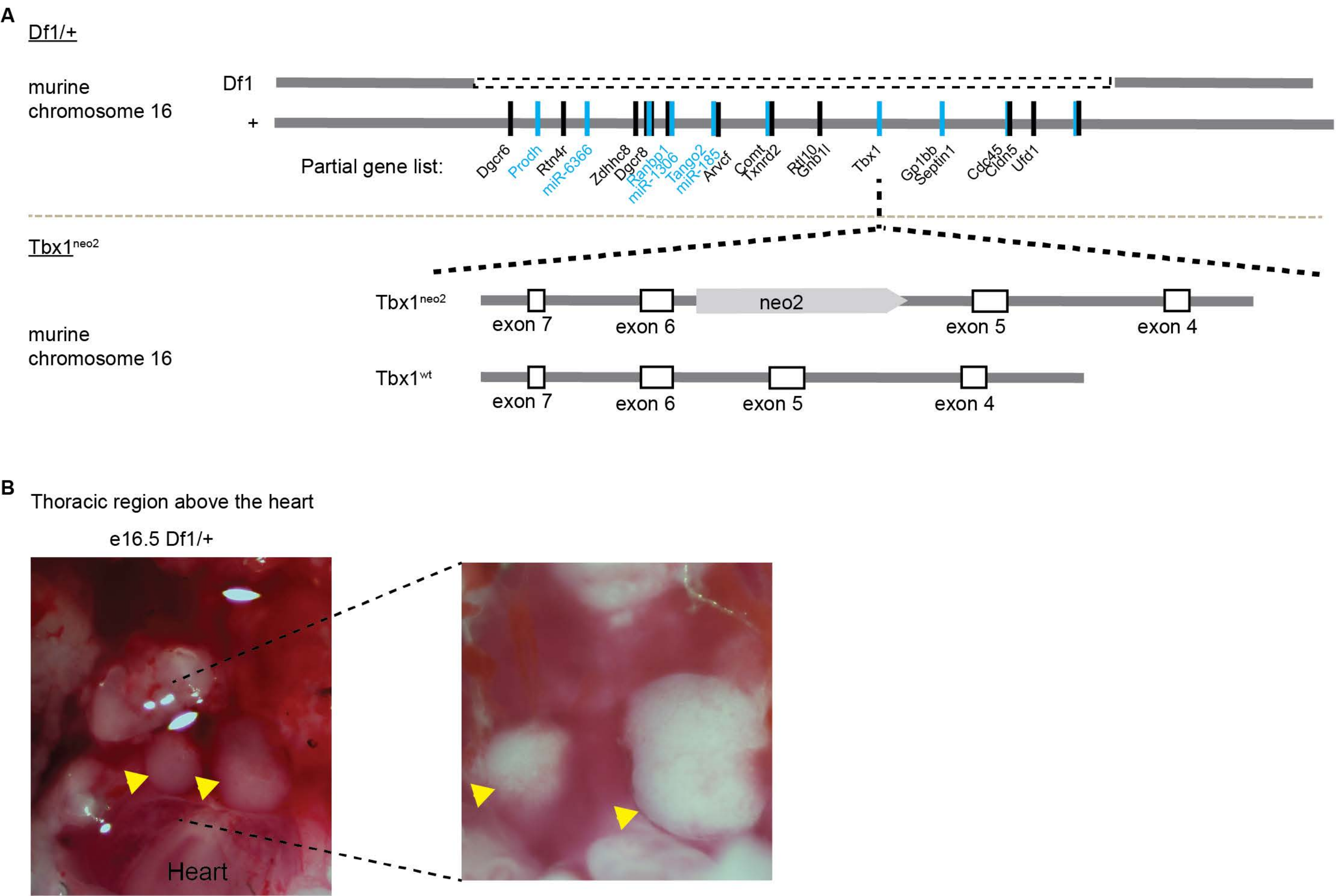
A
Df1/+
murine chromosome 16
Df1
+
Partial gene list:
Dgcr6
Prodh
Rtn4r
miR-6366
Zdhhc8
Dgcr8
Ranbp1
miR-1306
Tango2
miR-185
Arvcf
Comt
Txnrd2
Rtl10
Gnb1l
Tbx1
Gp1bb
Septin1
Cdc45
Cldn5
Ufd1
Tbx1neo2
murine chromosome 16
Tbx1neo2
neo2
exon 7
exon 6
exon 5
exon 4
Tbx1wt
exon 7
exon 6
exon 5
exon 4
B
Thoracic region above the heart
e16.5 Df1/+
Heart

Supplemental Fig. 2. **Human thymuses from 22q11.2DS patients have variable sizes while most retain normal T cell developmen**t. (**A**) Thymus tissue was obtained from children undergoing restorative cardiothoracic surgeries. The total size of the tissue was approximated based on surgical information, and a diagnosis of 22q11.2DS was subsequently established by genetic testing of the patients using either FISH or microarray. (**B**) Thymus tissue sections were prepared and processed for IHC. Antibodies against cytokeratin, EpCAM, CDR2 along with DAPI staining was used to visualize the indicated proteins. Merge represents an overall of the various fluorochromes. (**C**) T cell development was assessed by comparing the percentage of DN, DP, and mature SP subsets ($CD4^{+}CD8^{-}$ and $CD4^{-}CD8^{+}$). These subsets were distinguished by electronic gating after staining with antibodies selective for the human CD4 and CD8 cell surface proteins. The results are representative of 5 normal, 6 hypoplastic, and 3 aplastic tissues.

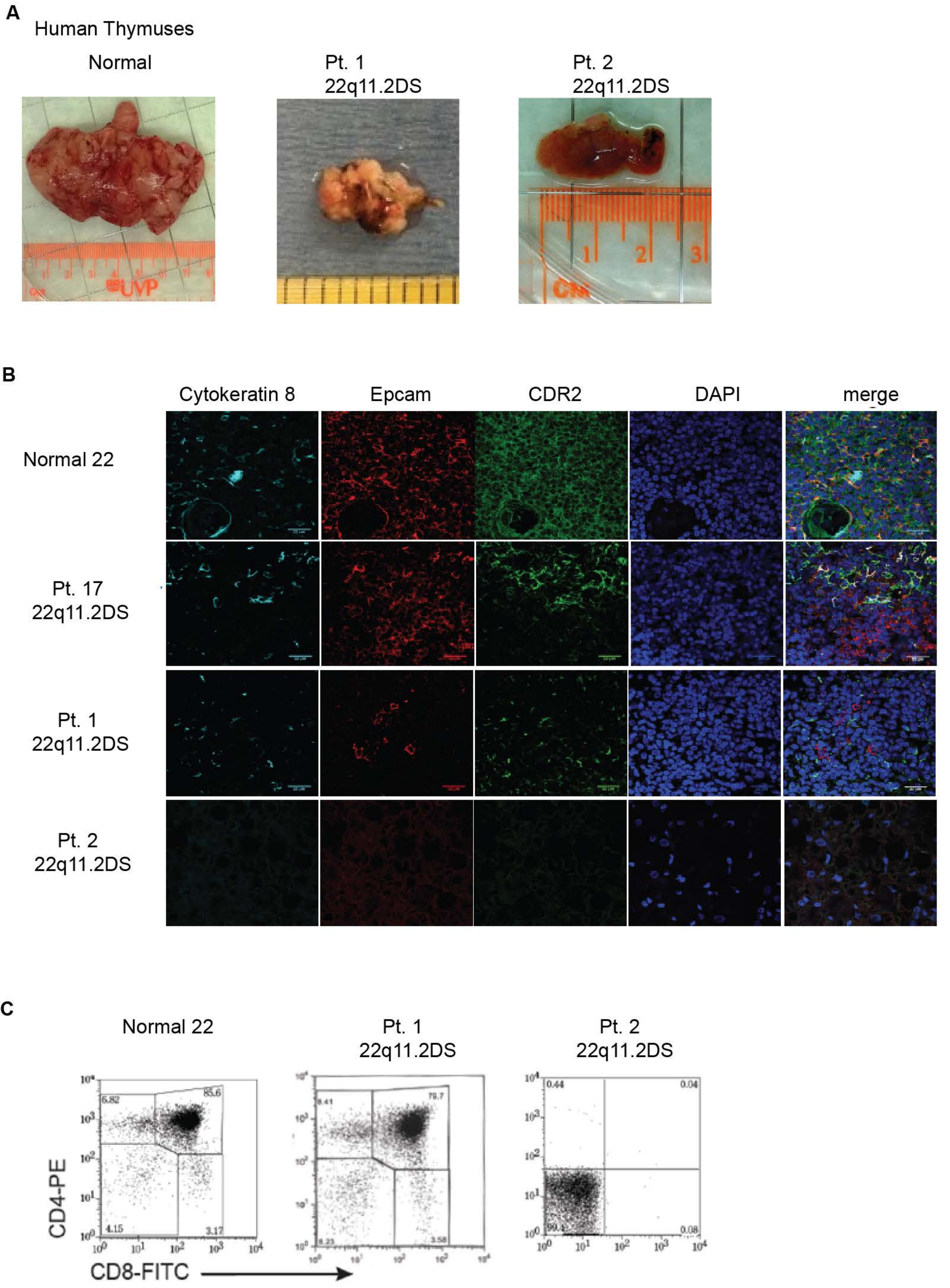
A
Human Thymuses
Normal
Pt. 1
22q11.2DS
Pt. 2
22q11.2DS
B
Cytokeratin 8
Epcam
CDR2
DAPI
merge
Normal 22
Pt. 17
22q11.2DS
Pt. 1
22q11.2DS
Pt. 2
22q11.2DS
C
Normal 22
Pt. 1
22q11.2DS
Pt. 2
22q11.2DS
CD4-PE
CD8-FITC
6.82
85.6
4.15
3.17
8.41
79.7
8.23
3.58
0.44
0.04
99.4
0.08

Supplemental Fig. 3. Cells from Tbx1$^{neo2/neo2}$ hypoplastic lobes have a similar small degree of cell death and proliferation. (**A**) Thymus lobes from the indicated genotypes were processed and the percent of cell death (7-AAD^{+}Annexin^{+}) and cell proliferation (Ki67^{+}) for mesenchymal cells (Mes), TECs and/or thymocytes is shown (Tbx1$^{+/+}$, Tbx1$^{+/neo2}$, Tbx1$^{neo2/neo2}$; n= 8, 14, 12, respectively). Statistically significant differences were established by ordinary one-way ANOVA.

Suppl. Fig 3

Day 4 FTOC

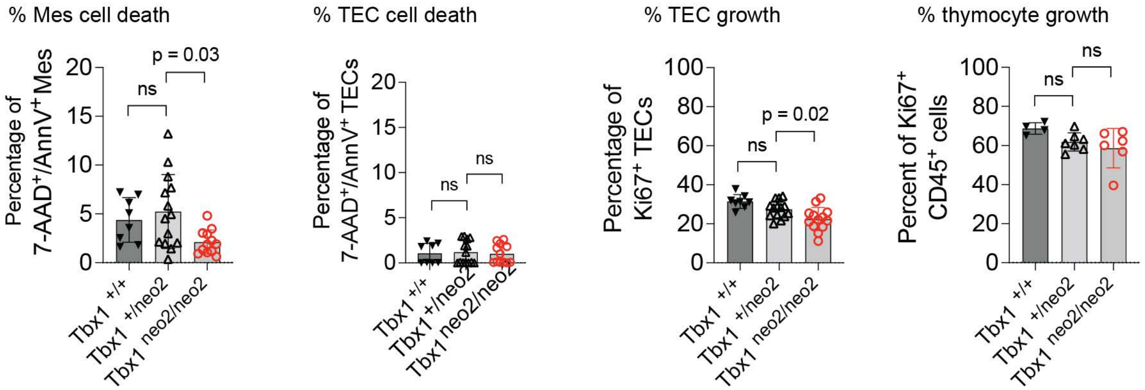

Supplemental Fig. 4. Normal thymus lobes placed around Tbx1$^{neo2/neo2}$ hypoplastic lobes fail to enable tissue expansion to the small tissues. (**A**) Paired e13-13.5 thymus lobes isolated from Tbx1$^{+/+}$ and Tbx1$^{neo2/neo2}$ embryos are placed onto 0.45 μm filters positioned on top of gel foam pads. In the 3rd column, a paired hypoplastic lobe is surrounded by 4 pairs of normal lobes. Column 4 reveals control paired thymus lobes surrounded by an additional 4 pairs of normal lobes. After 8- days of culture, live cell imaging reveals the changes in the lobes. The width of each rectangle on the grid is 3 mm, with the yellow bar = 1 mm. (**B-D**) Single cell suspensions from the centrally paired lobes are enumerated and assessed by flow cytometry. (**B**) The percentage of viable cells within the live cell gate is determined by forward (FSC) and side scatter (SSC) profiles using electronic gating. The boxed area represents the live cell gate. Results shown are representative of 6 independent experiments wherein the Tbx1$^{neo2/neo2}$ lobes dispersed and most cells died. (**C**) Flow cytometric analyses was performed on the cells isolated for thymocyte analyses. Antibodies against CD4, CD8, and the TCR beta subunit were used to assess thymocyte progression from the DN to DP and SP subsets. (**D**) The cell number per centrally located paired lobe is calculated. (Normal lobes, normal surround 4 N, and 22q11.2 hypo lobes around 4 normal; n = 4 independent experiments). Statistically significant differences were established by one-way ANOVA (Brown-Forsythe and Welch tests).

Suppl. Fig. 4

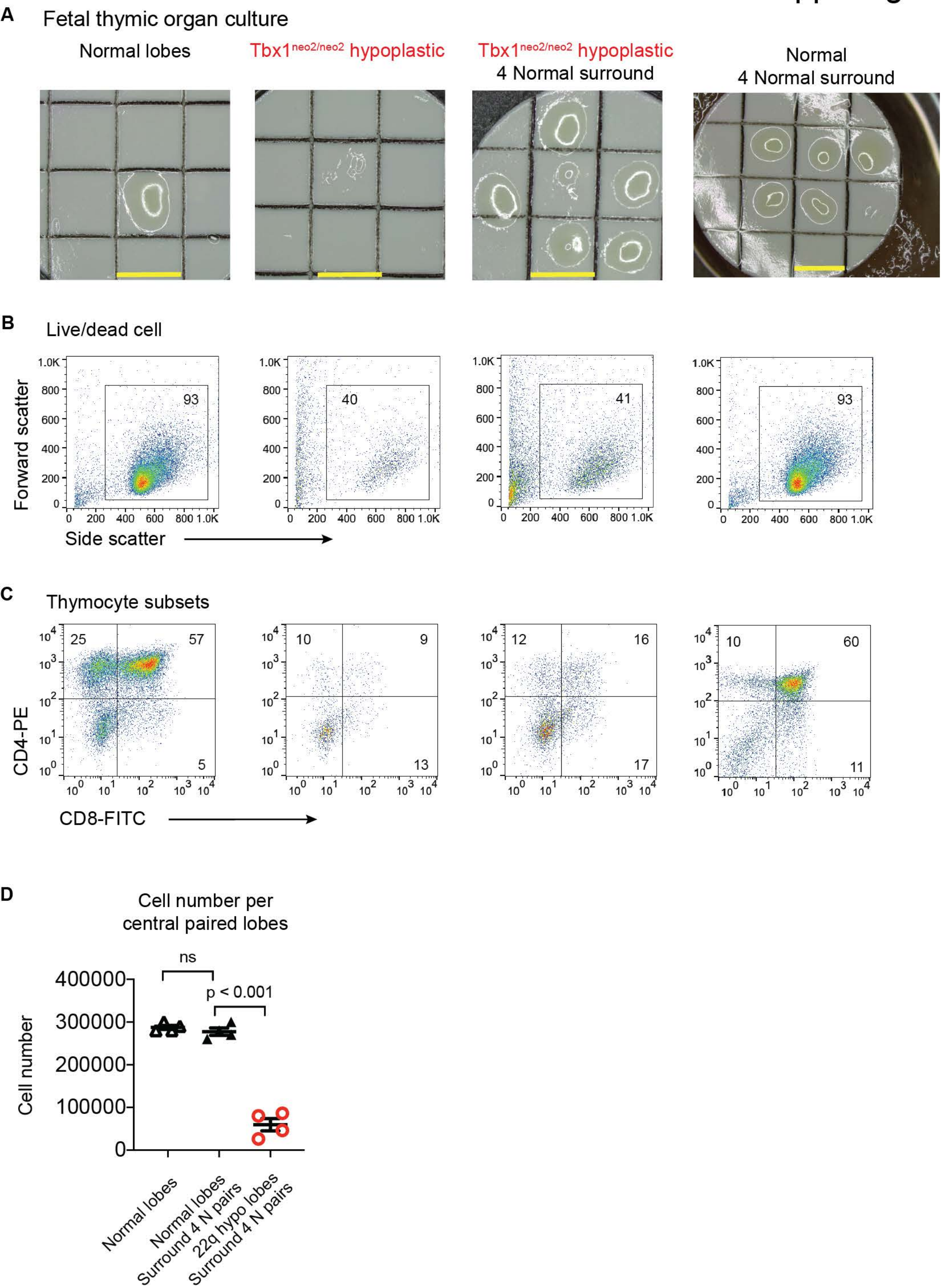
A
Fetal thymic organ culture
Normal lobes
Tbx1neo2/neo2 hypoplastic
Tbx1neo2/neo2 hypoplastic
4 Normal surround
Normal
4 Normal surround
B
Live/dead cell
Forward scatter
Side scatter
93
40
41
93
C
Thymocyte subsets
CD4-PE
CD8-FITC
25
57
5
10
9
13
12
16
17
10
60
11
D
Cell number per
central paired lobes
Cell number
ns
p < 0.001
400000
300000
200000
100000
0
Normal lobes
Normal lobes
Surround 4 N pairs
22q hypo lobes
Surround 4 N pairs

Supplemental Fig. 5. Reaggregate fetal thymic organ cultures require a minimum number of cells to support thymopoiesis. (**A**) Fetal thymic lobes were isolated from e13.0-13.5 embryos from timed pregnant C57Bl/6 mice. A pool of 8-12 lobes was digested with trypsin to generate a single cell suspension. The cells were stained with antibodies specific for mesenchymal cells (Pdgfra-PE) and thymus epithelial cells (EpCAM-FITC). As illustrated in Fig. 4A, the sorted Mes and TECs were pooled with the remaining $Pdgfra^{-}EpCam^{-}$ cell populations (ETPs and other hematopoietic cells and endothelial cells), maintaining the correct cell ratios as established with control thymuses. The total number of cells increased from 13700, 27400, 58400, and 109600 cells per aggregate. These were overlayed onto the filters, and RTOC continued for 10-days in culture. Live cell images are shown for the reaggregate cultures. In the images shown, the width of the yellow bar is 1 mm, with the entire boxed region measuring 3 mm. (**B-C**) Single cell suspensions were generated, and the cells analyzed by (**B**) live/dead cell gating and (**C**) cell surface staining of the CD4 and CD8 coreceptor molecules to determine the percentages of each of the thymocyte subsets (DN, DP, SP).

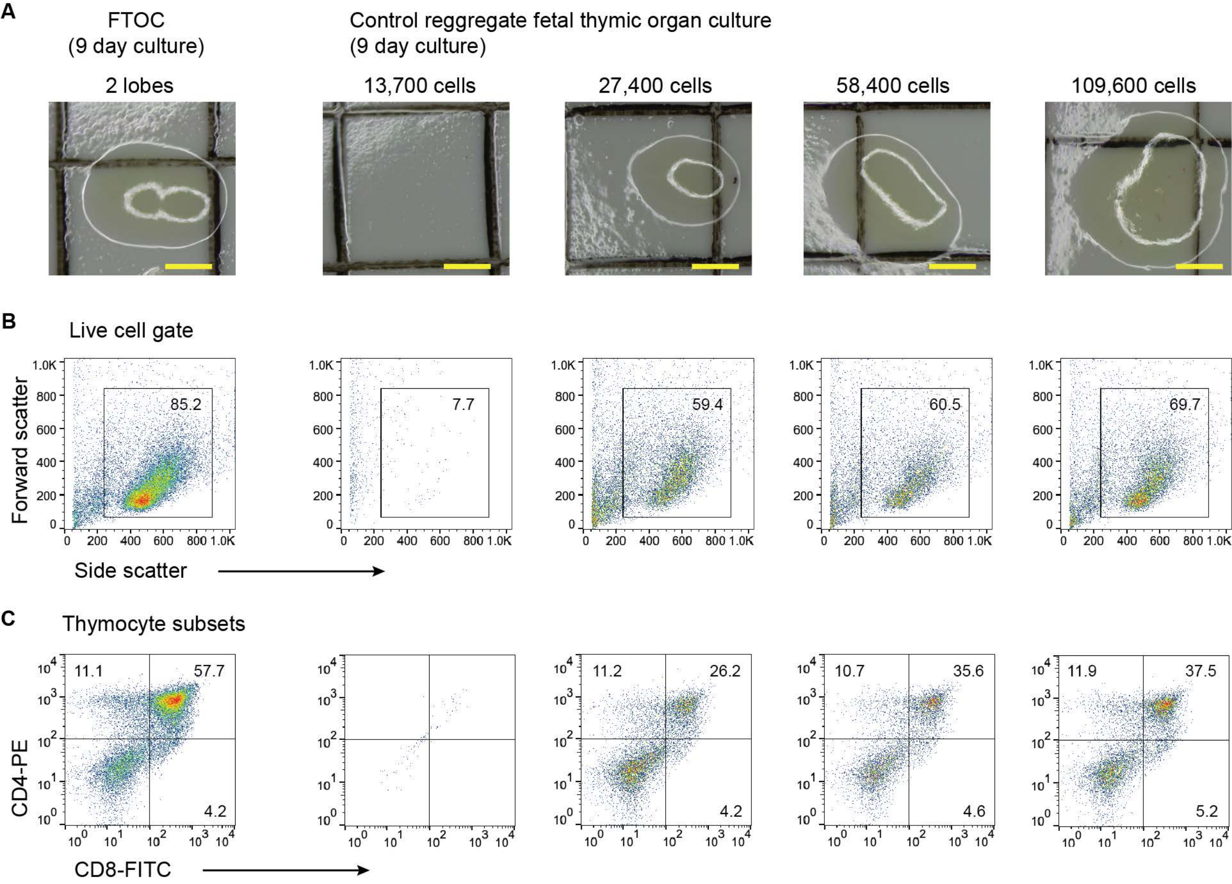
A
FTOC
(9 day culture)
Control reggregate fetal thymic organ culture
(9 day culture)
2 lobes
13,700 cells
27,400 cells
58,400 cells
109,600 cells
B
Live cell gate
Forward scatter
Side scatter
85.2
7.7
59.4
60.5
69.7
C
Thymocyte subsets
CD4-PE
CD8-FITC
11.1
57.7
4.2
11.2
26.2
4.2
10.7
35.6
4.6
11.9
37.5
5.2

Supplemental Figure 6. **$Tbx1^{neo2/neo2}$-derived mesenchymal cells do not negatively impact normal reaggregate thymus organ cultures**. (**A)**. Live cell imaging was used to visualize RTOCs after 10-days of culture. Control corresponds to the 3 subgroups of cells from $Tbx1^{+/+;+/neo2}$ thymus lobes. In the first column, control thymuses are a combination of cells from either $Tbx1^{+/+}$ and/or $Tbx1^{+/neo2}$ embryos. In the second column, 22q11.2DS hypoplastic thymuses were from $Tbx1^{neo2/neo2}$ embryos. In the third column, $Tbx1^{neo2/neo2}$ embryonic thymus sourced mesenchymal cells were used as substitutes for those in normal tissues (Sub normal Mes). Yellow bar = 1 mm. **(B)** Cell viability (upper row) and (**C**) thymopoiesis (DN to DP and then SP progression, lower row) were shown for the cells after 10-days of RTOC. (**D)** Cumulative cell numbers are shown for a representative RTOC experiment. (**E-G**) The fold increase in cell number is shown following 10-days of RTOC along with the cell viability and percentage of DP cells developing over this period. The number of independent experiments per group include $Tbx1^{+/+;+neo2}$ controls (n =17), $Tbx1^{neo2/neo2}$ (n = 16), and $Tbx1^{neo2/neo2}$ reconstituted normal (n =4). Statistical analyses done with One-Way ANOVA.

Suppl. Fig. 6

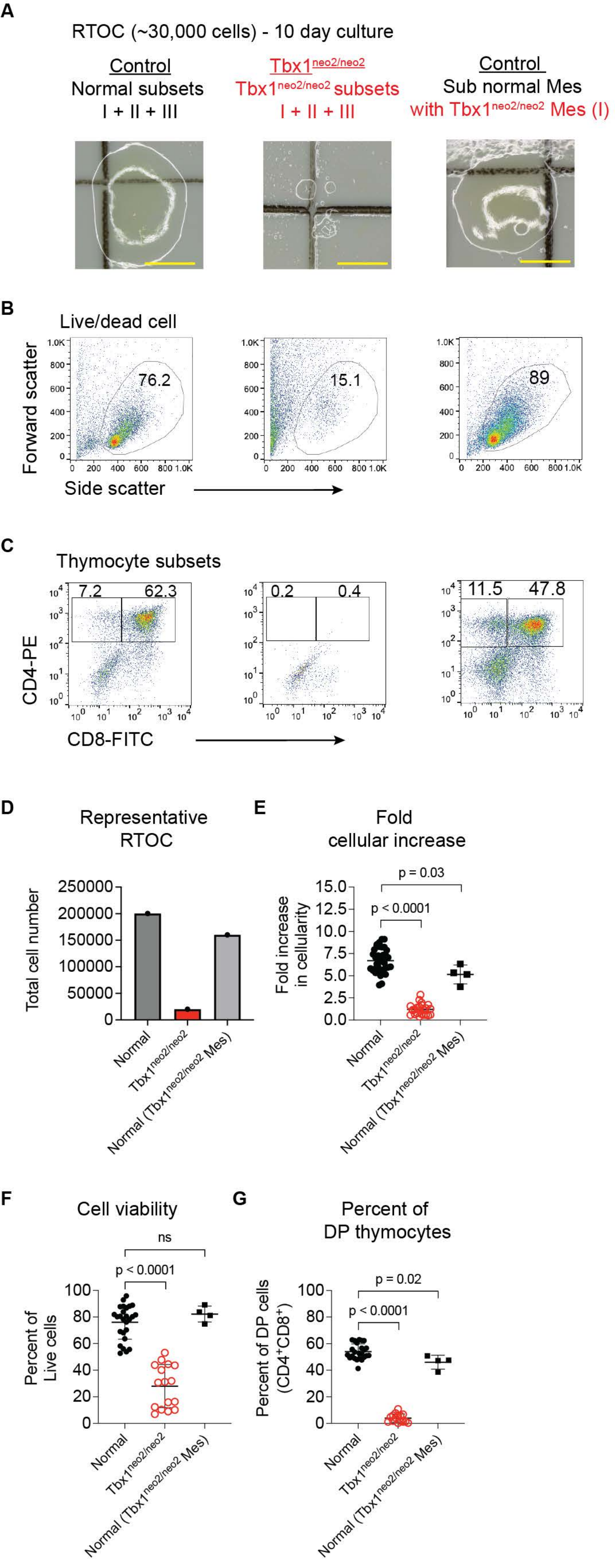

A
RTOC (~30,000 cells) - 10 day culture
Control
Normal subsets
I + II + III
Tbx1neo2/neo2
Tbx1neo2/neo2 subsets
I + II + III
Control
Sub normal Mes
with Tbx1neo2/neo2 Mes (I)
B
Live/dead cell
Forward scatter
Side scatter
76.2
15.1
89
C
Thymocyte subsets
CD4-PE
CD8-FITC
7.2
62.3
0.2
0.4
11.5
47.8
D
Representative
RTOC
Total cell number
Normal
Tbx1neo2/neo2
Normal (Tbx1neo2/neo2 Mes)
E
Fold
cellular increase
Fold increase
in cellularity
p = 0.03
p < 0.0001
F
Cell viability
Percent of
Live cells
ns
p < 0.0001
G
Percent of
DP thymocytes
Percent of DP cells
(CD4+CD8+)
p = 0.02
p < 0.0001

Supplemental Fig. 7. **Hypoplastic embryonic thymuses from *Foxn1* knock-in mice are blocked in early T cell development with a significant reduction in early thymus progenitors**. (**A**) Timed pregnancies were established by intercrossing Foxn1$^{wt/1089}$ heterozygous mice. Embryos from pregnant dams were isolated at e13-13.5. The cardiothoracic regions were accessed, and live cell images of the thymic lobes taken. Those from the Foxn1$^{1089/1089}$ genotype are hypoplastic relative to controls (Foxn1$^{+/+}$ and Foxn1$^{+/1089}$). Bar is 1 mm. (**B-C**) Flow cytometric analyses of single cell suspensions reveal the percentage of (**B**) mesenchymal (Pdgfra^{+}) and epithelial cells (EpCam^{+}), and (**C**) early thymus progenitors (ETPs), shown with the co-expression of CD117 (c-kit) and CD45. Included in this data set are the results from **Fig. 2**, co-presented here for comparative purposes. (**D**) The total thymus cell number and percentages of mesenchymal, epithelial, and early thymocyte progenitors (ETPs) are shown following with an analysis of Tbx1$^{+/+}$ (n = 17), Tbx1$^{+/neo2}$ (n = 32), Tbx1$^{neo2/+}$ (n = 28), Foxn1$^{+/1089}$ (n = 28) and Foxn1$^{1089/1089}$ genotyped embryos (n = 7). Some of the subset staining assays used fewer n values, as shown. Statistically significant differences were established by one-way ANOVA (Brown-Forsythe and Welch tests).

Suppl. Fig. 7

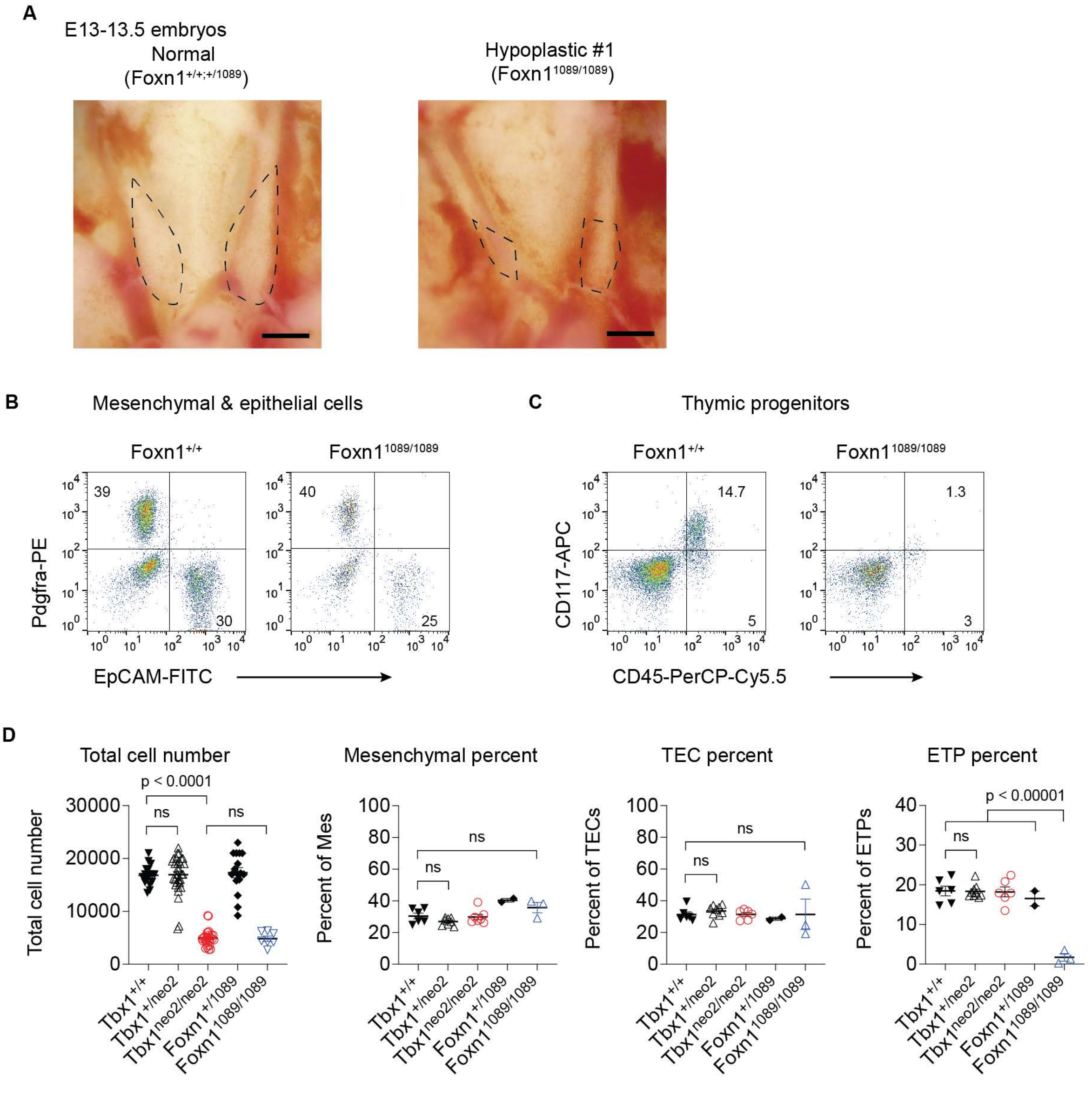
A
E13-13.5 embryos
Normal
(Foxn1+/+;+/1089)
Hypoplastic #1
(Foxn1 1089/1089)
B
Mesenchymal & epithelial cells
Foxn1+/+
Foxn1 1089/1089
Pdgfra-PE
EpCAM-FITC
39
30
40
25
C
Thymic progenitors
Foxn1+/+
Foxn1 1089/1089
CD117-APC
CD45-PerCP-Cy5.5
14.7
5
1.3
3
D
Total cell number
Mesenchymal percent
TEC percent
ETP percent
Total cell number
Percent of Mes
Percent of TECs
Percent of ETPs
p < 0.0001
p < 0.00001
ns
Tbx1+/+
Tbx1+/neo2
Tbx1neo2/neo2
Foxn1+/1089
Foxn1 1089/1089

Supplemental Fig. 8. Mesenchymal cell transcript alterations in Tbx1$^{neo2/neo2}$ embryonic thymuses. Single cell RNA sequencing reveals distinct transcript levels in the 5 mesenchymal cell subsets derived from embryonic thymuses from the control, Tbx1$^{neo2/neo2}$ and Foxn1$^{1089/1089}$ lines, displayed as violin plots. Expression level is shown on the y-axis.

Suppl. Fig. 8

Mesenchymal cell differences

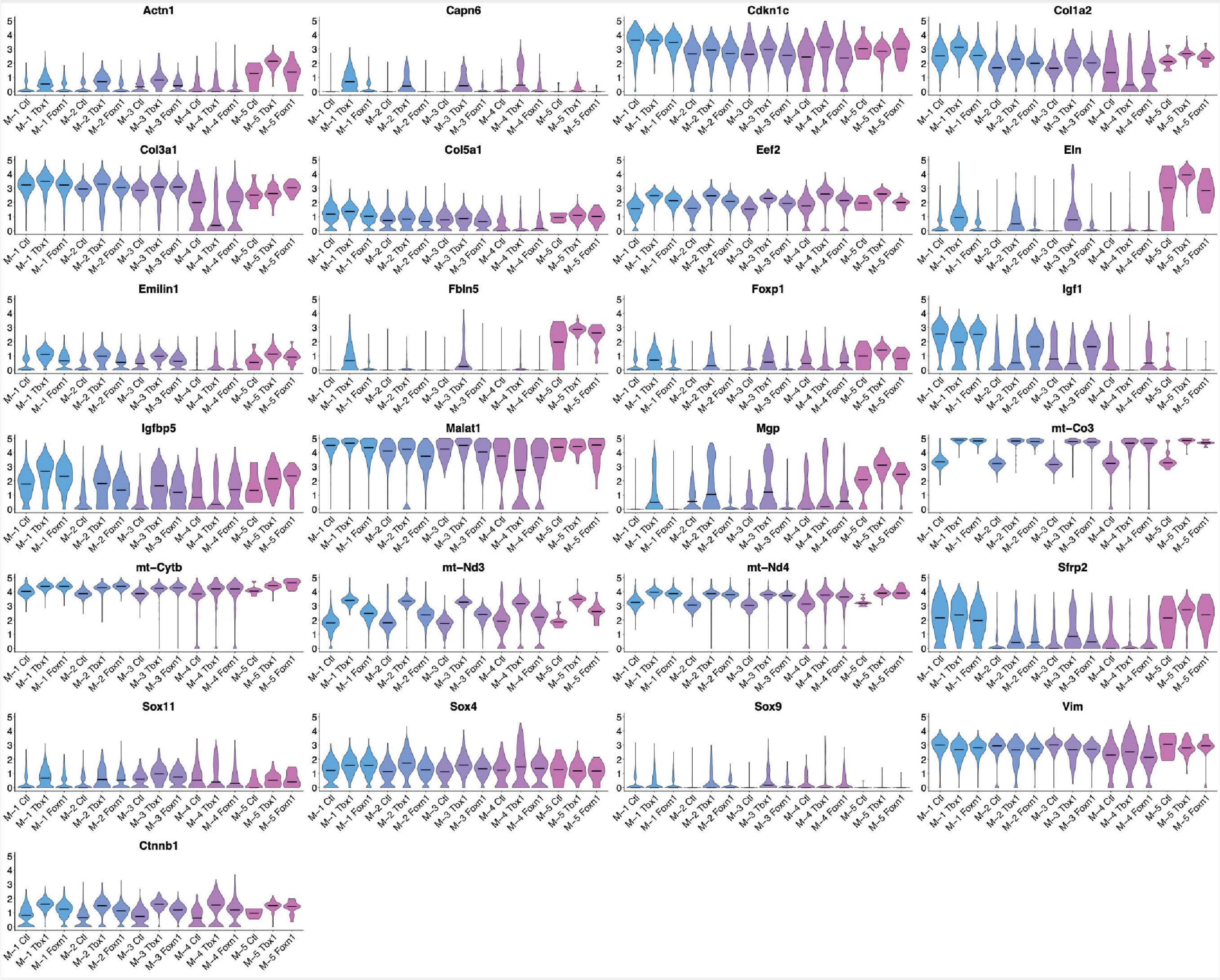

Supplemental Fig. 9. Small thymuses from the Df1/+ mouse model of 22q11.2DS exhibit a distinct RNA signature compared to normal sized thymuses. (**A**) Quantitative RT-PCR used to assess the levels of several genes previously reported critical for T cell development. This was done using paired left and right sided lobes from the indicated mice. This was done because some asymmetric development of the two lobes from the same embryo has been reported elsewhere. (**B)** RNA was prepared from e16.5 fetal thymuses derived from control and Df/1+ mice. RNA Sequencing was used to compare the transcriptomes of these cellular subsets. A heat map was used to indicate the up- (red) and down- (green) regulated genes from the indicated lobes. In the case of the Df/1+ embryo, a hypoplastic lobe and a paired normal sized lobe were compared. Transcripts comprising the genes haploinsufficient due to the chromosome 16 deletion were compared. On the right heat map are the differentially expressed genes uniquely identified in the hypoplastic lobe.

A

qRT-PCR of key genes in e16.5 control and Df1/+ thymuses

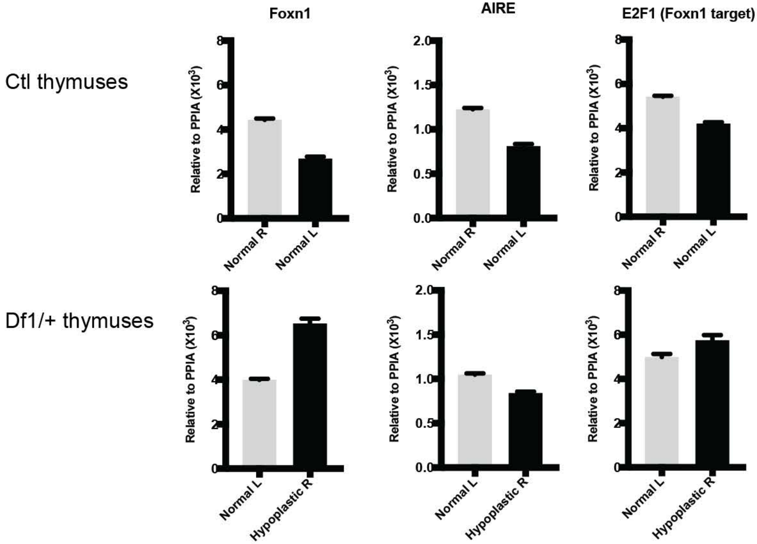

B

Transcript differences in e16.5 control and Df1/+ thymuses

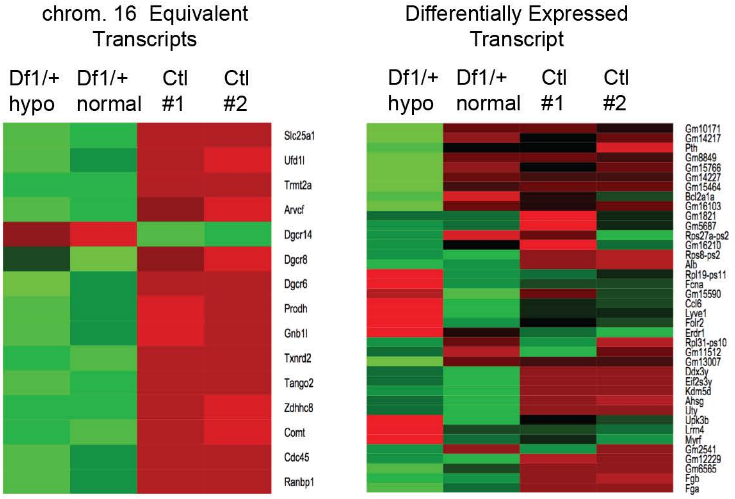

Supplemental Fig. 10. **Epithelial cell transcript alterations in Tbx1$^{neo2/neo2}$ embryonic thymuses**. Single cell RNA sequencing reveals distinct transcript levels in the 6 epithelial cell subsets derived from embryonic thymuses from the control, Tbx1$^{neo2/neo2}$ and Foxn1$^{1089/1089}$ lines, displayed as violin plots. Expression level is shown on the y-axis.

Epithelial cell differences

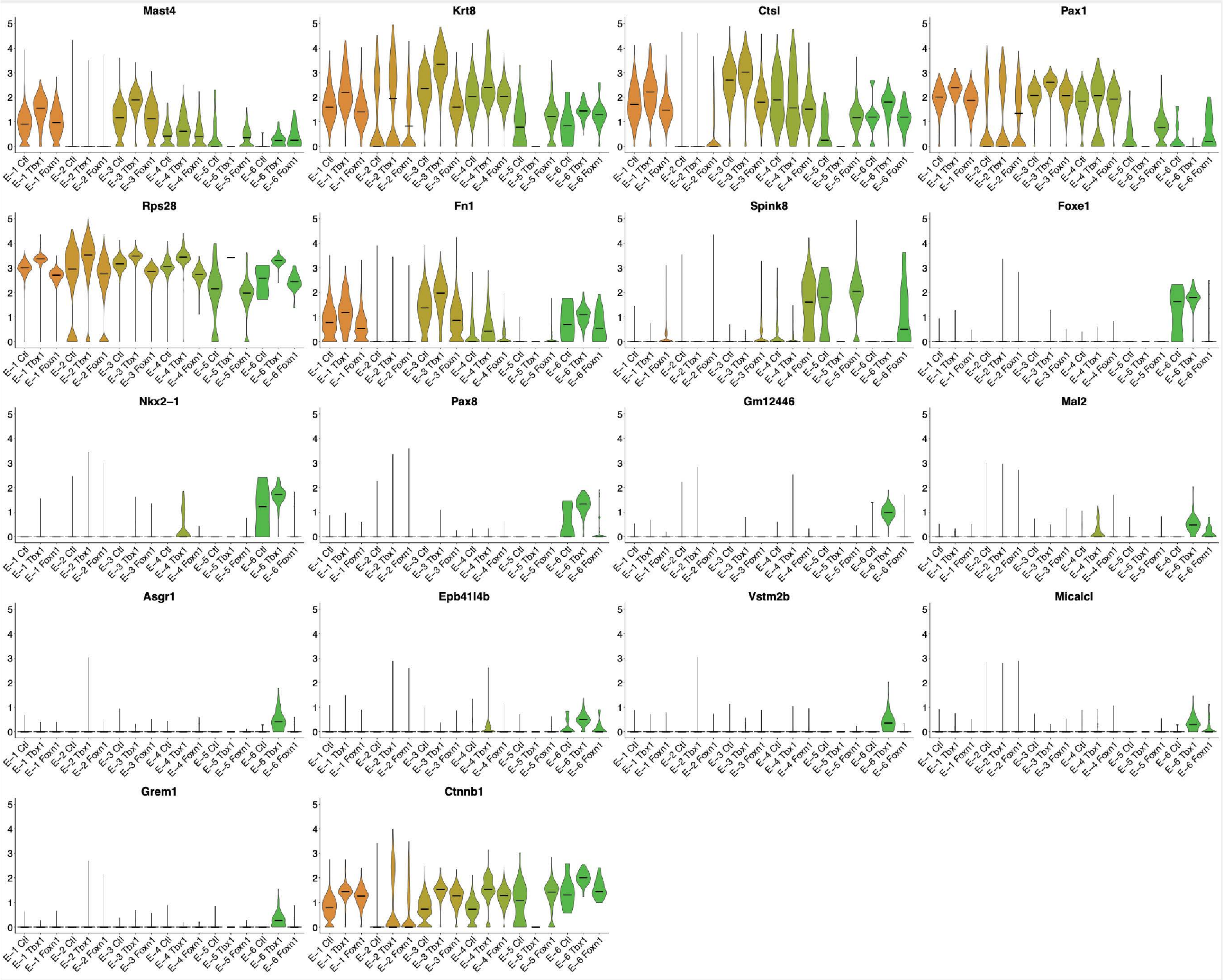

Supplemental Fig. 11. **Endothelial cell transcript alterations in Tbx1$^{neo2/neo2}$ embryonic thymuses**. Single cell RNA sequencing reveals distinct transcript levels in the one endothelial cell subset identified is each of the control, Tbx1$^{neo2/neo2}$ and Foxn1$^{1089/1089}$ thymuses. Violin plots were used to reveal the expression level differences. Expression level is shown on the y-axis.

Endothelial cell differences

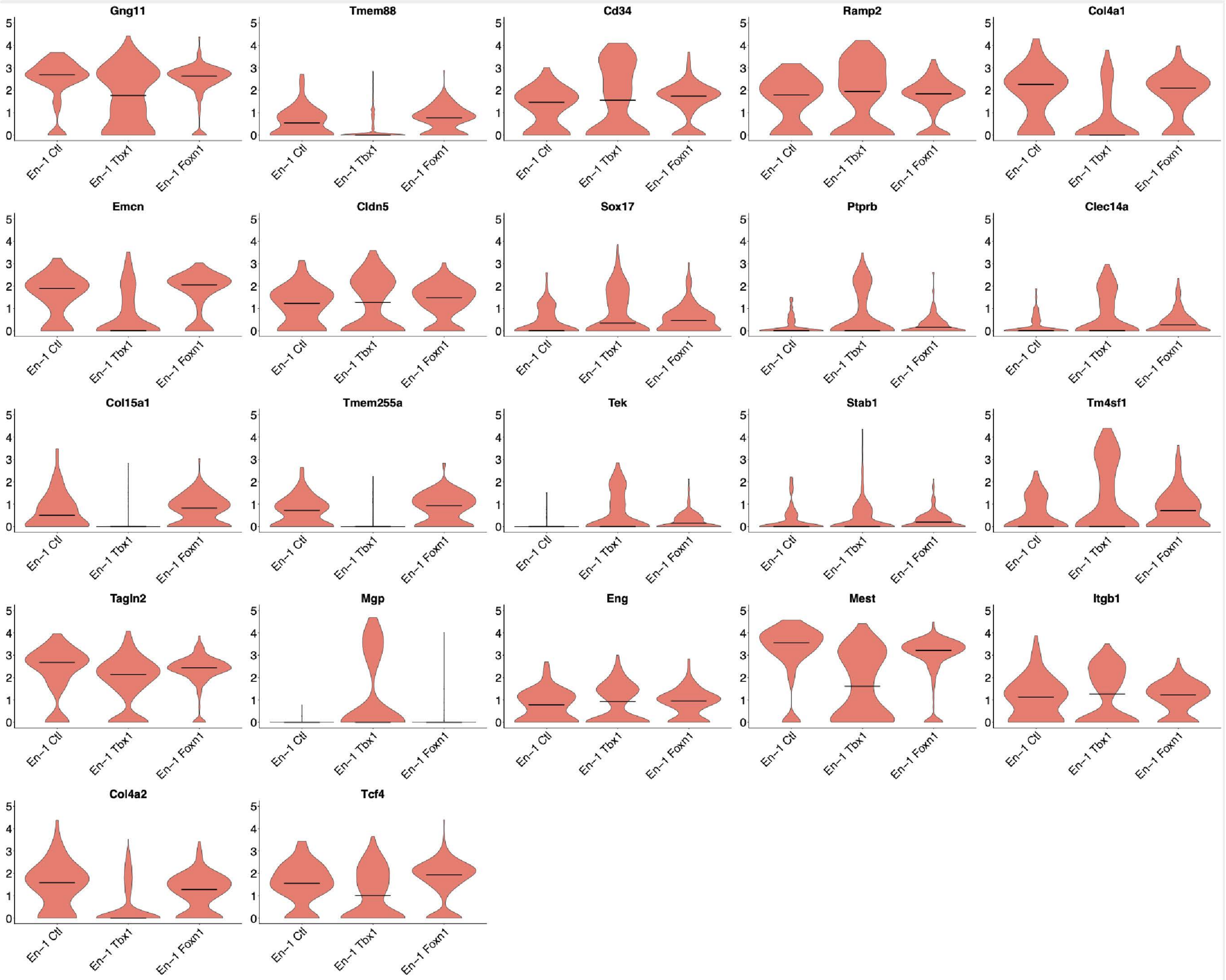

Supplemental Fig. 12. $Tbx1^{neo2/neo2}$ thymuses have a significantly increased percentage of $Pdgfrb^{+}Pdgfra^{-/lo}$ mesenchymal cells. (**A-B)** E13-13.5 embryonic thymuses from $Tbx1^{+/neo2}$ intercrossed time pregnant mice were genotyped and analyzed by flow cytometry. (**A**) Flow cytometric analyses of single cell suspensions reveal the percentage of mesenchymal cells that express Pdgfrb and/or Pdgfra. There is an over-representation of $Pdgfrb^{+}Pdgfra^{-/lo}$ cells in the $Tbx1^{neo2/neo2}$ embryonic thymuses. (**B**) the percentage of $Pdgfrb^{+}Pdgfra^{-/lo}$ mesenchymal cells in the normal sized and hypoplastic thymuses was determined using n = 5. Statistically significant differences were established by a Student's T-test.

Suppl. Fig. 12

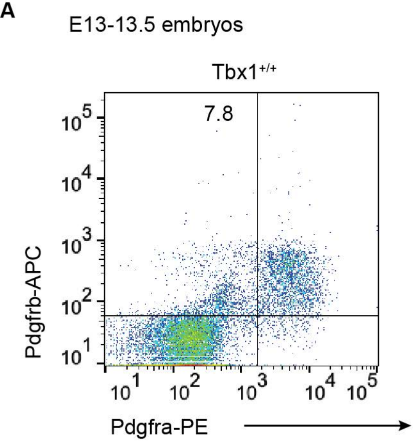
A
E13-13.5 embryos
Tbx1+/+
Tbx1neo2/neo2
7.8
25.4
Pdgfrb-APC
Pdgfra-PE
10^1 10^2 10^3 10^4 10^5

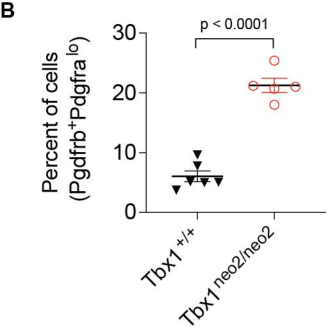
B
p < 0.0001
Percent of cells (Pgdfrb+Pdgfralo)
0
10
20
30
Tbx1 +/+
Tbx1 neo2/neo2

Supplemental Fig. 13. Human thymuses from 22q11.2DS patients have elevations in collagen relative to normal controls. Thymus tissues from two normal (#17 and #20) and three 22q11.2DS patients (#10, #19, #21) were obtained. Sections were prepared with tissue fragments and processed for IHC. Antibodies against CD31/Endomucin and Collagen were used for IHC, along with DAPI staining used to visualize the indicated proteins. Merge represents an overall of the various fluorochromes.

Suppl. Fig. 13

Post-natal human thymuses

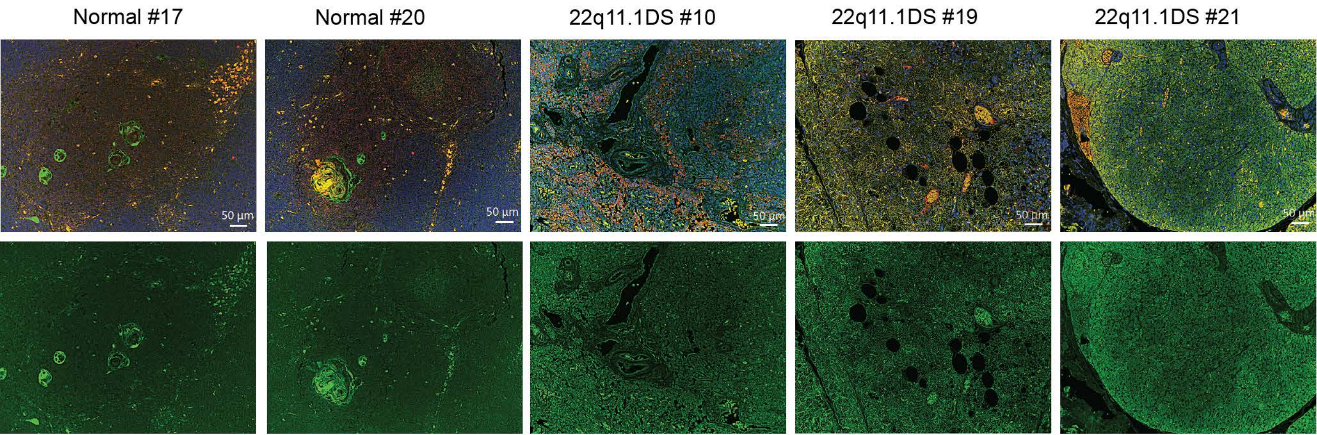

Collagen = green; CD31/Endomucin = Red, DAPI = blue

Supplemental Table 1. Comparative congenital malformations between human 22q11.2DS and mouse models

Chromosomal deletion or DNA mutation	Congenital Defects			
	TBX1 mRNA levels	Thymus phenotypes	Cardiac defects	Hypoparathyroidism or parathyroid anomalies
**Human 22q11.2 related deletion syndromes**				
22q11.2 deletions<br>(1.5 or 3 Mb deletions)	50%	60-70% hypoplasia[a]<br><1% aplasia	60-80% (ToF. IAA,VSD, and/or TA[b])	40%
TBX1 loss-of-function	100%	~30% hypoplasia	~30% (IAA, TA and/or VSD)	12%
TBX1 gain-of-function	100%	~30% hypoplasia	~30% (IAA,TA and/or VSD)	50%
**Mouse Models of 22q11.2DS**				
Df1/+ line<br>(1 Mb deletion on chromosome 16 – orthologous region of 22q11.2	50%	0-40% hypoplasia<br>(Penetrance is strain dependent)<br>0% on most mixed backgrounds	40% (IAA,TA, VSD and/or ARS[b])	100% on C57BL/6<br>Anomalous position of parathyroids<br>Reduced Parathyroid hormone levels
Del(3.0 Mb)/+<br>(3 Mb deletion on chromosome 16)	50%	47% with C57BL6 genetic knockin	20% IAA, ARS)	Not reported
$Tbx1^{neo2/neo2}$<br>(neo2 reverse orientation in intron 5)	34%	>90% hypoplasia<br><1% aplasia	>90% (IAA, VSD and/or ARS)	Not reported
$Tbx1^{neo2/neo}$<br>(neo2 as above, neo sense orientation in intron 5)	19%	70% aplasia<br>30% severe hypoplasia	100% (IAA, TA, VSD and/or ARS)	Not reported

[a]For most patients, thymus hypoplasia deduced from low TRECs at birth
[b]ToF = Tetralogy of Fallot; IAA = Interrupted aortic arch; TA = Truncus arteriosus; VSD = Ventricular septal defects, ARS = Aberrant right subclavian artery

**Supplemental Table 2**. Single cell RNA sequencing count summary				
Embryonic thymus genotype		**Control wt**	**Tbx1$^{neo2/neo2}$**	**Foxn1$^{1089/1089}$**
Estimated total # cells		6410	5759	12447
Mean reads/cell		76710	88877	40392
Median genes/cell		3157	4605	3404
Total # reads		491,715,368	511,845,934	502,760,800
Total genes detected		20,331	21,142	21,306
Cell Types	Thymus genotype	Number of cells/genotype		
		**Control wt**	**Tbx1$^{neo2/neo2}$**	**Foxn1$^{1089/1089}$**
	Total mesenchymal	1506 *(24.4)*[a]	2310 *(40.3)*	3575 *(29.7)*
	M1	634 *(42)*	966 *(42)*	1419 *(40)*
	M2	425 *(28.2)*	341 *(14.8)*	833 *(23.3)*
	M3	312 *(20.7)*	401 *(17.3)*	786 *(21.9)*
	M4	125 *(8.3)*	496 *(21.4)*	519 *(14.5)*
	M5	10 *(0.66)*	106 *(4.6)*	18 *(0.5)*
	Total epithelial	2524 *(41)*	1411 *(24.7)*	4945 *(41)*
	E1	1029 *(40.7)*	331 *(23.4)*	1227 *(24.8)*
	E2	479 *(19)*	582 *(41.2)*	1448 *(29.2)*
	E3	622 *(24.6)*	224 *(15.8)*	1189 *(24)*
	E4	347 *(13.7)*	170 *(12)*	661 *(13.3)*
	E5	40 *(1.59)*	2 *(0.14)*	393 *(7.9)*
	E6	7 *(0.2)*	102 *(7.2)*	27 *(0.54)*
	Endothelial	71 *(1.15)*	201 *(3.5)*	160 *(1.3)*
	Total hematopoietic	1578 *(25.6)*	593 *(10.3)*	720 *(6)*
	H1	1224 *(77.5)*	316 *(53.2)*	309 *(43)*
	H2	148 *(9.3)*	199 *(33.5)*	268 *(37.2)*
	H3	90 *(5.7)*	21 *(3.5)*	99 (13.8)
	H4	116 *(7.3)*	57 *(9.6)*	44 *(6.1)*
	Uncharacterized	485 *(7.8)*	1211 *(21)*	2625 *(21.8)*
	U1	347 *(71.5)*	364 *(30)*	2008 *(76.4)*
	U6	138 *(28.4)*	847 *(70)*	617 *(23.5)*

[a]Percentage of cells listed in the bracket

**Supplemental Table 3.** Top 20 gene identifiers for cell subsets							
Cluster Id	Cell Subset	Gene name	p_val	avg_logFC	pct.1	pct.2	p_val_adj
0	M-1	Col1a1	0	1.95077158	0.999	0.294	0
0	M-1	Sfrp2	0	1.92281881	0.922	0.195	0
0	M-1	Meg3	0	1.79249268	0.993	0.456	0
0	M-1	Col1a2	0	1.74209909	0.998	0.297	0
0	M-1	Igf1	0	1.7360948	0.908	0.208	0
0	M-1	Col3a1	0	1.68620084	0.997	0.356	0
0	M-1	Dcn	0	1.66408073	0.859	0.217	0
0	M-1	Ptn	0	1.65697197	0.999	0.64	0
0	M-1	Dlk1	0	1.55372675	0.98	0.343	0
0	M-1	Gpc3	0	1.49986808	0.987	0.417	0
0	M-1	Cdkn1c	0	1.46169121	0.995	0.649	0
0	M-1	Igfbp5	0	1.45493729	0.934	0.39	0
0	M-1	Postn	0	1.3823574	0.947	0.217	0
0	M-1	Cxcl12	0	1.35973389	0.888	0.299	0
0	M-1	Sparc	0	1.27921714	0.994	0.516	0
0	M-1	Lum	0	1.27637138	0.885	0.25	0
0	M-1	Mest	0	1.23381299	0.992	0.377	0
0	M-1	Mfap4	0	1.22636668	0.93	0.328	0
0	M-1	Akap12	0	1.209543	0.907	0.352	0
0	M-1	Peg3	0	1.17929513	0.979	0.438	0
1	U-1	Hba-a1	0	4.61365206	1	0.629	0
1	U-1	Hba-a2	0	4.60349063	1	0.582	0
1	U-1	Hbb-bs	0	4.57580034	0.999	0.798	0
1	U-1	Hbb-bt	0	4.51403823	1	0.651	0
1	U-1	Hbb-y	0	4.28740444	0.932	0.711	0
1	U-1	Hba-x	0	4.18618759	0.663	0.266	0
1	U-1	Hbb-bh1	0	2.4580798	0.485	0.031	0
1	U-1	Alas2	0	2.27480736	0.978	0.037	0
1	U-1	Nudt4	0	2.20218596	0.8	0.496	0
1	U-1	Snca	0	2.01984379	0.719	0.045	0
1	U-1	Bpgm	0	1.93769978	0.947	0.304	0
1	U-1	Mkrn1	0	1.78042906	0.851	0.401	0
1	U-1	Car2	0	1.66786055	0.753	0.074	0
1	U-1	Gypa	0	1.60199903	0.619	0.017	0
1	U-1	Blvrb	0	1.47377357	0.924	0.38	0
1	U-1	Ube2l6	0	1.36724687	0.908	0.08	0
1	U-1	Fth1	0	1.36398936	0.994	0.941	0
1	U-1	Gpx1	0	1.28039426	0.902	0.788	0
1	U-1	Fam46c	0	1.18791132	0.842	0.051	0
1	U-1	Fech	0	1.15605343	0.841	0.283	0
2	E-1	Epcam	0	1.07952316	0.99	0.308	0
2	E-1	Pax1	0	0.96364836	0.986	0.294	0
2	E-1	Pdgfa	0	0.95699698	0.985	0.355	0
2	E-1	Pltp	0	0.89974884	0.898	0.187	0
2	E-1	Pdlim1	0	0.87979196	0.967	0.376	0
2	E-1	H2afz	0	0.87689731	1	0.878	0
2	E-1	H2afx	0	0.87488391	0.987	0.609	0
2	E-1	Hist1h1b	0	0.85589779	0.764	0.268	0
2	E-1	Cenpf	0	0.8274075	0.88	0.256	0
2	E-1	Shisa2	0	0.82568751	0.952	0.239	0
2	E-1	Ube2c	0	0.77729566	0.89	0.437	0
2	E-1	Top2a	0	0.76749382	0.876	0.347	0
2	E-1	Hmga2	0	0.7673839	0.985	0.54	0
2	E-1	Limch1	0	0.75234517	0.888	0.217	0
2	E-1	Kcnn3	0	0.72948955	0.937	0.198	0
2	E-1	2810417H13Rik	0	0.72370582	0.982	0.456	0
2	E-1	Ifitm3	0	0.72344674	0.984	0.591	0
2	E-1	Hmgb2	0	0.71829438	0.998	0.753	0
2	E-1	Birc5	0	0.69775681	0.981	0.406	0
2	E-1	Il7	0	0.68897577	0.937	0.215	0
3	E-2	AY036118	5.74E-18	1.01301115	0.277	0.473	1.16E-13
3	E-2	Krt81	3.11E-109	0.92973402	0.521	0.365	6.30E-105
3	E-2	Gm42418	1.28E-17	0.91118286	0.583	0.889	2.58E-13
3	E-2	Lars2	3.93E-50	0.82490225	0.177	0.39	7.95E-46

3	E-2	Pax11	1.09E-110	0.79900655	0.515	0.352	2.21E-106
3	E-2	Hmgb21	3.41E-113	0.71208571	0.743	0.784	6.89E-109
3	E-2	Crabp21	6.73E-27	0.70092215	0.464	0.482	1.36E-22
3	E-2	Fabp51	7.61E-36	0.64618731	0.582	0.673	1.54E-31
3	E-2	Sfn1	2.78E-65	0.62467914	0.469	0.344	5.63E-61
3	E-2	Mif1	1.93E-176	0.58720761	0.825	0.835	3.91E-172
3	E-2	Pgam11	8.24E-71	0.58636534	0.668	0.791	1.67E-66
3	E-2	Krt181	1.76E-29	0.58464806	0.449	0.397	3.57E-25
3	E-2	Ptma1	0	0.55796887	0.984	0.946	0
3	E-2	S100a11	6.16E-91	0.54352265	0.739	0.824	1.25E-86
3	E-2	Pkm1	1.94E-84	0.53991256	0.715	0.806	3.92E-80
3	E-2	Dynll21	1.86E-14	0.52735942	0.377	0.624	3.76E-10
3	E-2	Gcat1	7.95E-10	0.52522979	0.299	0.458	1.61E-05
3	E-2	Snx31	7.28E-14	0.51369521	0.593	0.813	1.47E-09
3	E-2	Slc25a51	3.78E-14	0.5114453	0.565	0.739	7.65E-10
3	E-2	Rpl12	1.50E-133	0.4852237	0.844	0.931	3.03E-129
4	E-3	Ccl251	0	1.64392812	0.703	0.251	0
4	E-3	Krt182	0	1.62594903	0.947	0.352	0
4	E-3	Ctsl1	0	1.26212497	0.986	0.6	0
4	E-3	Krt82	0	1.22345399	0.983	0.326	0
4	E-3	Stmn22	0	1.17647625	0.971	0.423	0
4	E-3	Pltp1	0	1.13866052	0.903	0.204	0
4	E-3	Pax12	0	1.09318477	0.985	0.312	0
4	E-3	Prss161	0	1.02563506	0.445	0.116	0
4	E-3	Epcam1	0	1.01123843	0.977	0.326	0
4	E-3	Il72	0	0.99503192	0.942	0.233	0
4	E-3	Ifitm31	0	0.98004436	0.988	0.601	0
4	E-3	Psmb111	0	0.95437862	0.532	0.08	0
4	E-3	Pdlim12	0	0.95434009	0.952	0.392	0
4	E-3	Sfn2	0	0.9279526	0.981	0.299	0
4	E-3	Cd74	0	0.92082834	0.399	0.086	0
4	E-3	Nefm1	0	0.89897174	0.824	0.231	0
4	E-3	Hes62	0	0.8933074	0.9	0.384	0
4	E-3	Plgrkt1	0	0.88865449	0.912	0.487	0
4	E-3	Slc46a21	0	0.88856134	0.845	0.152	0
4	E-3	Hpgd1	0	0.88787301	0.883	0.27	0
5	H-1	Gzma	0	2.98354693	0.679	0.061	0
5	H-1	Ifitm1	0	2.79935834	0.975	0.284	0
5	H-1	Crip1	0	2.43914997	0.984	0.402	0
5	H-1	Plac8	0	2.20187992	0.983	0.212	0
5	H-1	Cmtm7	0	1.66984279	0.981	0.348	0
5	H-1	Nrgn	0	1.64778739	0.973	0.06	0
5	H-1	Rac2	0	1.64623604	0.982	0.057	0
5	H-1	Cd3g	0	1.63888989	0.515	0.016	0
5	H-1	Psmb8	0	1.63699896	0.975	0.047	0
5	H-1	Coro1a	0	1.6192326	0.979	0.072	0
5	H-1	Pim1	0	1.55923806	0.968	0.303	0
5	H-1	Ptprcap	0	1.50575862	0.972	0.024	0
5	H-1	Akr1c13	0	1.49443301	0.704	0.036	0
5	H-1	Ly6e	0	1.45201582	0.986	0.385	0
5	H-1	Srgn	0	1.45072567	0.976	0.052	0
5	H-1	Mzb1	0	1.44545305	0.834	0.023	0
5	H-1	Arhgdib	0	1.39156668	0.982	0.312	0
5	H-1	Tagln2	0	1.33914618	0.982	0.382	0
5	H-1	Ifitm21	0	1.23966541	0.968	0.722	0
5	H-1	Psme1	0	1.21459265	0.979	0.402	0
6	U-6	mt-Co3	6.91E-52	1.0405777	0.915	0.964	1.40E-47
6	U-6	mt-Nd41	5.37E-29	1.03047842	0.816	0.913	1.09E-24
6	U-6	mt-Cytb	1.75E-19	1.00099081	0.858	0.938	3.55E-15
6	U-6	mt-Nd2	1.83E-19	0.99632701	0.785	0.887	3.71E-15
6	U-6	mt-Nd31	3.32E-17	0.98756788	0.65	0.822	6.73E-13
6	U-6	H191	3.41E-43	0.98081641	0.633	0.731	6.90E-39
6	U-6	mt-Atp6	9.72E-18	0.96467736	0.912	0.962	1.97E-13
6	U-6	Igf21	6.87E-29	0.95492212	0.523	0.555	1.39E-24
6	U-6	Cdkn1c1	4.14E-149	0.91283043	0.77	0.687	8.37E-145
6	U-6	Lgals11	1.73E-125	0.85513022	0.769	0.725	3.51E-121

6	U-6	Vim1	3.00E-63	0.70960376	0.67	0.652	6.08E-59
6	U-6	Tpm21	4.64E-09	0.69439874	0.235	0.367	9.40E-05
6	U-6	Rbp11	1.21E-21	0.68905994	0.55	0.613	2.46E-17
6	U-6	mt-Nd51	1.21E-56	0.65356293	0.375	0.78	2.45E-52
6	U-6	mt-Nd4l1	2.64E-67	0.6399686	0.344	0.757	5.34E-63
6	U-6	Tuba1a1	7.51E-08	0.62090178	0.569	0.782	0.00152064
6	U-6	6330403K07Rik1	8.59E-11	0.59991228	0.265	0.432	1.74E-06
6	U-6	Nrep1	1.42E-33	0.58463027	0.247	0.5	2.88E-29
6	U-6	AY0361181	6.02E-41	0.54481131	0.216	0.469	1.22E-36
6	U-6	Tsc22d11	2.22E-16	0.53010993	0.348	0.61	4.50E-12
7	M-2	Mest1	0	1.28174994	0.984	0.417	0
7	M-2	Lum1	0	1.22425625	0.905	0.289	0
7	M-2	Dlk11	0	1.09124065	0.964	0.384	0
7	M-2	Col3a11	0	1.06353419	0.989	0.397	0
7	M-2	Peg32	0	0.95654921	0.964	0.473	0
7	M-2	Serpinh11	0	0.90438871	0.987	0.61	0
7	M-2	Lgals12	0	0.85197139	0.969	0.711	0
7	M-2	Fbn21	0	0.84881287	0.931	0.388	0
7	M-2	Ptn1	0	0.82732219	0.993	0.663	0
7	M-2	Rbp12	0	0.8191732	0.954	0.584	0
7	M-2	Tcf211	0	0.81237698	0.658	0.124	0
7	M-2	Itm2a1	0	0.8120336	0.927	0.436	0
7	M-2	Maged21	0	0.77333217	0.954	0.594	0
7	M-2	Vim2	0	0.76871468	0.966	0.631	0
7	M-2	Cd631	0	0.76357401	0.961	0.651	0
7	M-2	Igfbp41	0	0.75765566	0.974	0.55	0
7	M-2	Nr2f21	0	0.7571103	0.809	0.267	0
7	M-2	Igf22	0	0.75654565	0.98	0.523	0
7	M-2	Prrx12	0	0.75260104	0.856	0.251	0
7	M-2	Dcn1	0	0.74068543	0.77	0.264	0
8	M-3	Mest2	0	1.20845751	0.992	0.419	0
8	M-3	Lum2	0	1.1325516	0.929	0.291	0
8	M-3	Top2a2	0	1.08829058	0.905	0.371	0
8	M-3	Cenpa1	0	1.08538533	0.845	0.351	0
8	M-3	Cenpf1	0	1.04909679	0.893	0.286	0
8	M-3	Col3a12	0	1.04677113	0.995	0.4	0
8	M-3	Hist1h2ap2	0	1.02880383	0.743	0.34	0
8	M-3	Mki671	0	1.02874842	0.936	0.305	0
8	M-3	Birc51	0	1.02565784	0.989	0.433	0
8	M-3	Peg33	0	0.99261908	0.985	0.474	0
8	M-3	2810417H13Rik2	0	0.98347015	0.987	0.482	0
8	M-3	Cks22	0	0.97677084	0.985	0.515	0
8	M-3	Tpx21	0	0.94216808	0.937	0.31	0
8	M-3	Ube2c2	0	0.92961025	0.951	0.455	0
8	M-3	Smc21	0	0.92332754	0.973	0.475	0
8	M-3	Arl6ip12	5.70E-271	0.90804418	0.944	0.638	1.15E-266
8	M-3	H2afx1	0	0.90272608	0.991	0.627	0
8	M-3	Dlk12	0	0.8953643	0.966	0.387	0
8	M-3	Cenpe1	0	0.88732011	0.863	0.255	0
8	E-4	Prc11	0	0.8857983	0.81	0.248	0
9	E-4	Krt5	0	2.87983304	0.882	0.092	0
9	E-4	Krt17	0	2.25566213	0.529	0.062	0
9	E-4	Krtdap	0	1.97293452	0.733	0.075	0
9	E-4	Gstm11	0	1.9615075	0.761	0.266	0
9	E-4	Clu1	0	1.78993338	0.865	0.201	0
9	E-4	Sfn3	0	1.70802758	0.987	0.325	0
9	E-4	Perp2	0	1.5299558	0.963	0.25	0
9	E-4	Dsp2	0	1.34372323	0.948	0.248	0
9	E-4	Spink8	0	1.34258055	0.623	0.081	0
9	E-4	Krt183	0	1.30002665	0.993	0.372	0
9	E-4	Fxyd33	0	1.22967281	0.96	0.259	0
9	E-4	Epcam2	0	1.1427078	0.986	0.35	0
9	E-4	S100a14	0	1.0925645	0.636	0.063	0
9	E-4	Krt83	0	1.09061826	0.992	0.35	0
9	E-4	Wfdc22	0	1.05847303	0.871	0.246	0
9	E-4	Cldn7	0	1.02646405	0.872	0.135	0

9	E-4	Hspb11	0	1.02305152	0.745	0.243	0
9	E-4	Cebpb	0	0.99542073	0.926	0.412	0
9	E-4	Pdcd41	0	0.98423146	0.97	0.632	0
9	E-4	Cd9	0	0.97438257	0.968	0.383	0
10	M-4	Mgp2	1.77E-30	1.24910107	0.534	0.407	3.59E-26
10	M-4	Itm2a3	7.89E-85	1.02132201	0.682	0.459	1.60E-80
10	M-4	Sparc3	5.36E-60	0.8095389	0.727	0.569	1.08E-55
10	M-4	Tuba1a4	7.74E-71	0.66658682	0.867	0.763	1.57E-66
10	M-4	Col1a13	8.59E-31	0.65782014	0.571	0.373	1.74E-26
10	M-4	Capn62	4.10E-87	0.64990669	0.46	0.236	8.30E-83
10	M-4	Sox111	1.04E-87	0.63942172	0.548	0.311	2.11E-83
10	M-4	Cdkn1c4	2.78E-122	0.63774508	0.9	0.683	5.63E-118
10	M-4	Ccnd1	2.22E-37	0.58588	0.378	0.23	4.49E-33
10	M-4	Nnat4	1.48E-79	0.58407184	0.659	0.421	2.99E-75
10	M-4	Col1a23	3.08E-49	0.57288703	0.639	0.372	6.24E-45
10	M-4	Vim4	1.19E-114	0.57090074	0.91	0.64	2.41E-110
10	M-4	Tpm24	2.75E-25	0.53614081	0.496	0.351	5.56E-21
10	M-4	H194	2.49E-66	0.52381895	0.838	0.719	5.04E-62
10	M-4	Sox41	6.70E-12	0.52231647	0.713	0.686	1.36E-07
10	M-4	Aldh1a22	1.97E-46	0.51753394	0.282	0.138	3.99E-42
10	M-4	Dpysl3	1.46E-25	0.51169296	0.387	0.258	2.96E-21
10	M-4	Ube2c3	9.27E-15	0.50289221	0.554	0.483	1.88E-10
10	M-4	Cks23	9.82E-15	0.50036538	0.626	0.54	1.99E-10
10	M-4	Fbln21	2.22E-54	0.48571638	0.337	0.165	4.50E-50
11	H-2	Apoe	7.46E-234	4.00310213	0.715	0.296	1.51E-229
11	H-2	Pf4	0	3.40004844	0.472	0.027	0
11	H-2	C1qb	0	2.95090285	0.634	0.01	0
11	H-2	C1qc	0	2.67292483	0.577	0.007	0
11	H-2	C1qa	0	2.63315077	0.554	0.007	0
11	H-2	Fcer1g1	0	2.60297955	0.714	0.11	0
11	H-2	Tyrobp1	0	2.28368323	0.681	0.108	0
11	H-2	Mt12	3.68E-211	2.24985656	0.641	0.214	7.44E-207
11	H-2	Aif1	0	2.20532874	0.6	0.012	0
11	H-2	Sepp11	1.10E-98	2.18094554	0.543	0.29	2.22E-94
11	H-2	Ctsb	1.23E-41	2.16919216	0.553	0.539	2.49E-37
11	H-2	Tmsb4x2	1.66E-281	2.16408403	0.976	0.932	3.35E-277
11	H-2	Hmox1	1.42E-60	2.15252143	0.363	0.153	2.87E-56
11	H-2	Sat1	2.41E-164	2.11695231	0.644	0.302	4.88E-160
11	H-2	Lyz2	0	2.06236657	0.496	0.022	0
11	H-2	Ctsd	1.44E-50	2.01829558	0.564	0.499	2.92E-46
11	H-2	Ckb1	5.42E-200	1.73308585	0.629	0.221	1.10E-195
11	H-2	Lgmn	4.81E-69	1.70423594	0.468	0.261	9.74E-65
11	H-2	Ms4a7	0	1.69550253	0.433	0.003	0
11	H-2	Ctss	0	1.65498818	0.452	0.02	0
12	E-5	Pth	0	5.74675332	0.995	0.267	0
12	E-5	Chga	0	4.90446753	0.966	0.121	0
12	E-5	Ccl21a1	0	3.79591482	0.949	0.086	0
12	E-5	Spp1	0	3.36130377	0.685	0.041	0
12	E-5	Npnt	0	2.97341122	0.892	0.199	0
12	E-5	Mafb	0	2.93151949	0.936	0.249	0
12	E-5	Sparcl12	0	2.50627142	0.878	0.209	0
12	E-5	Ibsp	0	2.4354863	0.871	0.016	0
12	E-5	Gnas	2.40E-271	2.36794097	0.995	0.936	4.87E-267
12	E-5	Rnase41	0	2.34559331	0.862	0.167	0
12	E-5	Pla2g7	0	2.32919974	0.855	0.081	0
12	E-5	Gdpd1	0	2.28562211	0.878	0.137	0
12	E-5	Kansl1l	0	2.08313149	0.871	0.212	0
12	E-5	Gng12	5.12E-276	1.98297987	0.892	0.34	1.04E-271
12	E-5	Id4	0	1.9295697	0.88	0.134	0
12	E-5	Spink81	0	1.88164494	0.871	0.094	0
12	E-5	Ece1	0	1.82265114	0.834	0.207	0
12	E-5	Ddx26b	0	1.80325364	0.839	0.162	0
12	E-5	Cited2	1.03E-245	1.72304014	0.885	0.369	2.08E-241
12	E-5	Fam20a	0	1.7154901	0.805	0.037	0
13	En-1	Gng11	2.46E-302	2.24219122	0.843	0.234	4.97E-298
13	En-1	Fabp4	0	2.1954431	0.583	0.024	0

13	En-1	Egfl7	7.99E-238	1.98478198	0.771	0.249	1.62E-233
13	En-1	Cd342	4.38E-179	1.92050585	0.678	0.225	8.86E-175
13	En-1	Ramp23	5.14E-207	1.9187837	0.711	0.219	1.04E-202
13	En-1	Cav1	0	1.8505242	0.72	0.03	0
13	En-1	Cdh5	0	1.82640413	0.66	0.007	0
13	En-1	Emcn	0	1.70829495	0.63	0.021	0
13	En-1	S100a161	0	1.70638766	0.771	0.139	0
13	En-1	Crip2	7.98E-205	1.70337084	0.785	0.305	1.61E-200
13	En-1	Tm4sf12	2.20E-161	1.69065633	0.549	0.131	4.45E-157
13	En-1	Cldn5	0	1.689682	0.681	0.002	0
13	En-1	Lmo21	0	1.67511305	0.72	0.062	0
13	En-1	Pecam1	0	1.54902644	0.653	0.062	0
13	En-1	Tagln23	7.54E-170	1.54780087	0.843	0.421	1.53E-165
13	En-1	Sox18	0	1.53643009	0.727	0.005	0
13	En-1	Prkcdbp	3.11E-257	1.53043192	0.748	0.194	6.30E-253
13	En-1	Col4a13	1.51E-55	1.51585965	0.602	0.401	3.05E-51
13	En-1	Vim5	2.71E-184	1.51521558	0.951	0.647	5.49E-180
13	En-1	Ecscr	0	1.42277435	0.632	0.026	0
14	H-3	Ccl5	0	3.085397	0.567	0.02	0
14	H-3	Ifitm11	1.17E-135	2.75235639	0.922	0.332	2.36E-131
14	H-3	Gzma1	4.73E-241	2.62177662	0.77	0.103	9.59E-237
14	H-3	Cd522	0	2.44143649	0.876	0.095	0
14	H-3	Gzmb1	8.24E-154	2.18577822	0.429	0.046	1.67E-149
14	H-3	Tyrobp2	0	2.17931288	0.982	0.115	0
14	H-3	Fcer1g2	0	1.90525272	0.977	0.118	0
14	H-3	Cd3g1	0	1.85341648	0.935	0.046	0
14	H-3	Srgn2	0	1.79686031	0.986	0.115	0
14	H-3	Crip11	4.17E-115	1.76317706	0.977	0.442	8.44E-111
14	H-3	Cd71	0	1.74545186	0.834	0.049	0
14	H-3	Rgs11	0	1.73655792	0.876	0.066	0
14	H-3	Ikzf22	9.02E-217	1.72578084	0.972	0.234	1.83E-212
14	H-3	Klrd1	0	1.72412815	0.433	0.009	0
14	H-3	Rac22	0	1.72185194	0.977	0.121	0
14	H-3	Ptprcap1	0	1.7081882	0.995	0.089	0
14	H-3	Cpa31	0	1.70412145	0.747	0.06	0
14	H-3	Lmo42	2.15E-47	1.70078842	0.843	0.522	4.35E-43
14	H-3	Coro1a2	0	1.69650453	0.986	0.135	0
14	H-3	Itga41	6.70E-297	1.6695363	0.912	0.121	1.36E-292
15	H-4	Cd742	3.63E-246	2.99947132	0.786	0.107	7.35E-242
15	H-4	Cd523	0	2.85883673	0.995	0.094	0
15	H-4	Cst33	1.05E-88	2.47635538	0.976	0.7	2.13E-84
15	H-4	Tyrobp3	0	2.46668405	0.981	0.116	0
15	H-4	H2-Aa1	3.25E-242	2.42074276	0.595	0.059	6.58E-238
15	H-4	Alox5ap3	0	2.14114472	0.929	0.112	0
15	H-4	Napsa2	0	2.08015685	0.871	0.076	0
15	H-4	Coro1a3	0	2.05536206	0.986	0.135	0
15	H-4	Lsp12	1.24E-185	2.03405191	0.962	0.276	2.51E-181
15	H-4	H2-Eb12	8.57E-169	1.98732456	0.567	0.077	1.74E-164
15	H-4	Ifi302	4.70E-107	1.96834508	0.824	0.321	9.51E-103
15	H-4	Ckb3	1.66E-121	1.96668975	0.776	0.226	3.36E-117
15	H-4	Fcer1g3	0	1.83520212	0.957	0.118	0
15	H-4	Arhgdib3	2.11E-147	1.81182277	0.967	0.358	4.28E-143
15	H-4	Gm2a3	1.07E-187	1.7901777	0.881	0.197	2.17E-183
15	H-4	Anxa11	1.84E-101	1.78131504	0.467	0.077	3.73E-97
15	H-4	Rac23	0	1.75649489	0.976	0.121	0
15	H-4	Plac82	3.46E-131	1.72612496	0.905	0.266	7.01E-127
15	H-4	Cd72	0	1.61232472	0.738	0.05	0
15	H-4	Ifi203	0	1.60572608	0.652	0.023	0
16	E-6	Foxe1	0	1.67379076	0.809	0.004	0
16	E-6	Hhex3	7.54E-262	1.55096104	0.816	0.073	1.53E-257
16	E-6	Nkx2-1	0	1.53935829	0.809	0.009	0
16	E-6	Wfdc24	9.07E-111	1.50975247	0.949	0.273	1.84E-106
16	E-6	Mt13	6.74E-100	1.36485534	0.882	0.221	1.36E-95
16	E-6	Prlr1	0	1.35905857	0.809	0.025	0
16	E-6	Mt21	3.99E-268	1.35016274	0.801	0.066	8.08E-264
16	E-6	Pax8	0	1.19206522	0.809	0.004	0

16	E-6	Bex43	5.29E-82	1.14526029	0.926	0.375	1.07E-77
16	E-6	Slc16a2	1.70E-194	1.11495387	0.831	0.105	3.45E-190
16	E-6	Cldn72	3.71E-162	1.11026712	0.963	0.167	7.50E-158
16	E-6	Epcam4	3.83E-65	1.02818686	1	0.378	7.76E-61
16	E-6	Mbip	5.83E-95	1.00843309	0.882	0.274	1.18E-90
16	E-6	Cd24a2	8.87E-60	0.98367881	0.971	0.664	1.80E-55
16	E-6	Iyd	0	0.9699743	0.728	0.003	0
16	E-6	Cldn62	1.64E-169	0.95380307	0.949	0.159	3.31E-165
16	E-6	Bex13	1.08E-82	0.93071084	0.949	0.338	2.18E-78
16	E-6	Gm12446	0	0.88608395	0.765	0.003	0
16	E-6	Itga62	2.13E-131	0.86303835	0.882	0.177	4.32E-127
16	E-6	Kcnk11	2.89E-132	0.86185265	0.904	0.19	5.84E-128
17	M-5	Eln2	5.37E-168	3.32644612	0.978	0.193	1.09E-163
17	M-5	Acta21	2.68E-251	3.17990926	0.955	0.11	5.43E-247
17	M-5	Tagln	1.12E-268	2.936248	0.97	0.105	2.26E-264
17	M-5	Fbln51	1.10E-234	2.44534721	0.978	0.126	2.22E-230
17	M-5	Rgs5	0	2.17591124	0.903	0.067	0
17	M-5	Myl92	1.31E-111	1.90782143	0.97	0.292	2.66E-107
17	M-5	Mgp4	5.88E-84	1.8954859	0.963	0.41	1.19E-79
17	M-5	Actn11	1.39E-100	1.65953189	0.97	0.365	2.81E-96
17	M-5	Actg2	0	1.60718308	0.754	0.023	0
17	M-5	Tm4sf13	9.96E-205	1.58555261	0.963	0.134	2.02E-200
17	M-5	Bgn4	1.53E-127	1.58244683	0.993	0.272	3.10E-123
17	M-5	Sfrp22	1.67E-96	1.55886109	0.97	0.283	3.38E-92
17	M-5	Fbn13	3.23E-124	1.39700361	0.985	0.261	6.54E-120
17	M-5	Tpm26	1.64E-93	1.37452566	0.985	0.355	3.33E-89
17	M-5	Sparc6	2.39E-73	1.34085357	1	0.574	4.84E-69
17	M-5	H196	5.32E-60	1.28761792	0.993	0.723	1.08E-55
17	M-5	Cald16	6.64E-71	1.28760228	0.993	0.559	1.34E-66
17	M-5	Jag11	1.54E-104	1.28341097	0.97	0.299	3.11E-100
17	M-5	Fn13	9.37E-80	1.24915304	0.993	0.447	1.90E-75
17	M-5	Col4a15	1.82E-86	1.2242529	0.985	0.401	3.68E-82

**Supplemental Table 4**: Significantly altered pathways in mesenchymal subsets comparing Tbx1$^{neo2/neo2}$ and Foxn1$^{1089/1089}$ to wildtype controls										
**Canonical Pathways Affected**	**Mesenchymal cell subset**									
	M1		M2		M3		M4		M5	
Respective Mutants normalized to WT	Tbx1	Foxn1	Tbx1	Foxn1	Tbx1	Foxn1	Tbx1	Foxn1	Tbx1	Foxn1
Wnt/β-catenin Signaling	3.25[a]	1.88	6.26	1.12	4.75	0.56	5.13	2.08	3.62	0.41
Hepatic Fibrosis Signaling Pathway	4.96	1.39	2.49	1.06	4.12	1.89	5.28	0.83	0.94	0.42
ILK Signaling	8.07	1.98	2.47	3.69	3.71	4.17	5.10	1.84	2.22	0.69
IGF-1 Signaling	1.54	1.31	1.47	1.73	1.83	0.85	4.49	3.79	1.33	0.00
VEGF Signaling	0.91	0.41	0.43	0.73	0.67	0.22	2.08	1.80	0.45	0.27
Integrin Signaling	4.20	0.99	1.47	2.39	2.65	1.80	2.68	2.20	0.94	0.36
IL-8 Signaling	3.04	0.64	1.12	0.69	2.16	1.00	1.91	0.87	0.54	0.38
PI3K/AKT Signaling	0.44	1.06	0.71	1.79	0.72	0.54	2.01	3.93	0.66	0.40
Regulation of the Epithelial-Mesenchymal Transition Pathway	1.71	0.50	2.07	1.22	2.35	1.40	1.73	1.18	0.54	0.14
Remodeling of Epithelial Adherens Junctions	2.48	4.24	2.49	2.63	4.45	4.71	4.41	2.55	1.04	0.37
Actin Cytoskeleton Signaling	2.31	0.73	0.29	1.25	1.13	1.40	2.27	0.96	2.00	0.45
Gap Junction Signaling	0.61	1.54	0.40	1.16	0.93	2.46	2.63	1.40	0.54	0.38
Tight Junction Signaling	1.77	0.63	0.80	1.50	1.03	0.81	2.87	0.46	1.23	0.28
Regulation of the Epithelial-Mesenchymal Transition Pathway	1.71	0.50	2.07	1.22	2.35	1.40	1.73	1.18	0.54	0.14
HOTAIR Regulatory Pathway	1.93	0.47	3.87	0.73	3.71	1.14	3.05	0.71	0.85	1.48

[a]Values are negative log of p value, higher the value more significant is the pathway. We have used 1.3 (p value 0.05) as cutoff.

**Supplemental Table 5**. Reagents and supplies used in the study		
REAGENT or RESOURCE	SOURCE	IDENTIFIER
Antibodies		
Pdgfra (Gt)	R and D Systems	Cat# AF1062 RRID:AB_2236897
E cadherin (Ms)	BD Biosciences	Cat# 610182, RRID:AB_397581
Laminin (Rb)	Sigma-Aldrich	Cat# L9393, RRID:AB_477163
EpCAM (Rb)	Thermo Fisher Scientific	Cat# PA5-19832, RRID: AB_10984102
Pdgfrb (Rb)	Cell Signaling Technology	Cat# 3169, RRID:AB_2162497
Cytokeratin 8 (Ms)	BioLegend	Cat# 904804, RRID:AB_2616821<br>Clone 1E8
Cytokeratin 14 (Rb)	DB Biotech	Cat# DB 099-0.1<br>Lot# DB099-0.1-02B
Pan cytokeratin (Ms)	Thermo Fisher Scientific	Cat# MA1-82041, RRID:AB_2281092
Collagen I (Rb)	Abcam	Cat# ab21286
Collagen I (Rb)	Abcam	Cat# ab138492
Endomucin (Rat)	Santa Cruz	Cat# sc-65495
CD31/Pecam1 (Rat)	BD Biosciences	Cat# BD553370
Mcam/CD146 (Rb)	Abcam	Cat# ab75769
Cspg4/Ng2 (Ms)	Millipore-Sigma	Cat# MAB2029
Smooth Muscle Actin (Ms)	R and D Systems	Cat# MAB1420, RRID:AB_262054
Donkey Anti-Rabbit IgG H&L (Alexa Fluor® 488)	Abcam	Cat# ab150073 RRID:AB_2636877
Donkey Anti-Rabbit IgG H&L (Alexa Fluor® 594)	Abcam	Cat# ab150076, RRID:AB_2782993
Donkey Anti-Mouse IgG H&L (Alexa Fluor® 488)	Abcam	Cat# ab150105, RRID:AB_2732856
Donkey Anti-Mouse IgG H&L (Alexa Fluor® 594)	Abcam	Cat# ab150108, RRID:AB_2732073
Donkey Anti-Goat IgG H&L (Alexa Fluor® 594)	Abcam	Cat# ab150132, RRID:AB_2810222
Donkey Anti-Rabbit IgG H&L (Alexa Fluor® 647)	Abcam	Cat# ab150075, RRID:AB_2752244
Anti-CD8-FITC	Tonbo Biosciences	Cat# 35-0081, RRID:AB_2621671
Anti-CD4-PE	Thermo Fisher Scientific	Cat# 50-0041-80,RRID:AB_10596360
Anti-TCR-b-PerCP-Cy5.5	Tonbo Biosciences	Cat# 65-5961, RRID:AB_2621911
Anti-CD69-APC	Thermo Fisher Scientific	Cat# 17-0691-82, RRID:AB_1210795
Anti-CD45-PerCPCy5.5	BioLegend	103132 Clone 30-F11
Anti-B220-APC	Tonbo Biosciences	Cat# 20-0452, RRID:AB_2621574
Anti-CD44-APC	BD bioscience	Cat# 559250, RRID:AB_398661
Anti-CD25-FITC	BD bioscience	Cat# 553072, RRID:AB_394604
Anti-CD8-PE	Tonbo Biosciences	Cat# 50-0081, RRID:AB_2621741
Anti-B220-PE	BD bioscience	Cat# 553090, RRID:AB_394620
Anti-NK1.1-PE	BD bioscience	Cat# 553165, RRID:AB_394677
Anti-γδTCR-PE	BD bioscience	Cat# 553178, RRID:AB_394689
Anti-CD11b-PE	BD bioscience	Cat# 557397, RRID:AB_396680
Anti-CD11c-PE	Thermo Fisher Scientific	Cat# 50-0114-80, RRID:AB_11151322
Anti-CD19-PE	Thermo Fisher Scientific	Cat# 50-0193-82, RRID:AB_11218286
Anti-Ter-119-PE	Tonbo Biosciences	Cat# 50-5921, RRID:AB_2621802
EpCAM-FITC	BioLegend	Cat# 118207, RRID:AB_1134106
PDGFRa- PE	Thermo Fisher Scientific	Cat# 12-1401-81, RRID:AB_657615
CD117-APC	Tonbo Biosciences	Cat# 20-1172, RRID:AB_2621587
Anti-Annexin V-APC	Biolegend	Cat# 640920
7-AAD-viability staining	eBiosciences	Cst# 00-6993-50
Anti-Ki67-APC	Invitrogen	Cat# 17-5698-82
Anti-CD45-APC Cy7	BD Pharmingen	Cat# 561037
Anti-EpCAM-APC	Invitrogen	Cat# 17-5791-80
Anti-UEA-1-PE	Vector Laboratories	Cat# RL-1062, Lot Z0314
Anti-Ly-51-FITC	BD Pharmingen	Cat# 553160
Anti-CD31-PerCPCy5.5	BioLegend	Cat#102522
Bacterial and Virus Strains		

BL21 Codon plus	Thermo-Fisher Scientific	Cat#C602003
Biological Samples		
Human thymuses	See IRB	
Embryos	C57BL/6 mice	
Thymus	C57BL/6 mice	
Tails	C57BL/6 mice	
Chemicals, Peptides, and Recombinant Proteins		
DAPI	Thermo Fisher Scientific	Cat# D1306, RRID:AB_2629482
Shandon Immu-Mount	Thermo Fisher Scientific	Cat# 9990402
Trypsin	Sigma	Cat# T4049
Minoxidil	Sigma	Cat# M4145
Verteporfin	Sigma	Cat# SML0534
Critical Commercial Assays		
MesenCult™ Expansion Kit (Mouse)	StemCell Technologies	Cat#05513
Prolong Gold Anti-fade Montant	Thermo-Fisher Scientific	Cat#P10144
EpiCult™ Expansion Kit (Mouse)	StemCell Technologies	Cat#06070
Verteporfin	Thomas Scientific	Cat#C816P07
Minoxidil	Sigma	Cat#M4145
Beta- amino propionitrile	Sigma	Cat#A3134
Prostaglandin E2	Sigma	Cat#538904
Deposited Data		
GEO -scRNA Seq	170686	
Experimental Models: Organisms/Strains		
Df1/+ line (Del(16Es2el-Ufd1l)217Bld)	Stanislav S. Zakharenko	St. Judes' Research Hospital
$Tbx1^{+/neo2}$ line	Antonio Baldini	CNR Institute of Genetics and Biophysics, Naples
C57Bl/J	Jackson Labs	Cat # 000664
PDGFRα-eGFP	Jackson Labs	Cat # 007669
$Foxn1^{1089/1089}$	van Oers lab	$Foxn1^{1089}$
Oligonucleotides		
RT PCR Primers, Genotyping Primers		
Tbx1 neo2 GCCAGAGGCCACTTGTGTAG	This paper	#1869
Tbx1neo2 GGAGGTCTTCTGGTTTACCCT	This paper	#1868
Tbx1neo2 AGGCTGGGATTCCAAAAGAC	This paper	#1867
Murine Aire 5' TGCATAGCATCCTGGACGGCTTCC	This paper	#1356
Murine Aire 3' CCTGGGCTGGAGACGCTCTTTGAG	This paper	#1357
Murine E2F1 5' TAGCCCTGGGAAGACCTCAT	This paper	#1263
Murine E2F1 3' CCCCAAAGTCACAGTCAAAGAG	This paper	#1264
Murine Foxn1 5' CCAGGGCCACTGCACAGCCGGACC	This paper	#1062
Murine Foxn1 3' CAAGTGCCATGGCCGTCTGGGCC	This paper	#1063
Murine Plod1 F GAAGGATGACGCCAAGCTAGA	This paper	#2117
Murine Plod1 R TGAAGAACTGAGCTGAACGCT	This paper	#2118
Murine Plod2 F GAGAGGCGGTGATGGAATGAA	This paper	#2119

Murine Plod2 R ACTCGGTAAACAAGATGACCAGA	This paper	#2120
Murine Col1a1 F GCTCCTCTTAGGGGCCACT	This paper	#2121
Murine Col1a1 R CCACGTCTCACCATTGGGG	This paper	#2122
Murine Col1a2 F GTAACTTCGTGCCTAGCAACA	This paper	#2123
Murine Col1a2 R CCTTTGTCAGAATACTGAGCAGC	This paper	#2124
Murine Gapdh F AGGTCGGTGTGAACGGATTTG	This paper	#1583
Murine Gapdh R TGTAGACCATGTAGTTGAGGTCA	This paper	#1584
Software and Algorithms		
Microsoft Office 365	Microsoft	
GraphPad Prism	GraphPad Software, Inc.	Prism 9.1
SnapGene	GSL Biotech LLC	SnapGene 5.2.4
PyMol molecular visualization software	Schrodinger, Inc.	PyMol 2.4
Keyence BT software	Keyence Corp.	BT W
FlowJo flow cytometry software	TreeStar Inc.	Flowjo 10.6.1
Image J	NIH source software	https://imagej.nih.gov/ij/
Cell ranger	10X Genomics	Cell Ranger 3.0.0

Excel Data File 1. Differentially expressed genes identified among all 17 cell subsets identified using scRNA comparing control, Tbx1$^{neo2/neo2}$ and Foxn1$^{1089/1089}$ embryonic day 13-13.5 thymuses.

Movie 1. The movie reveals the blood flow through the aorta with an interrupted aortic arch type B in one of the Tbx1$^{neo/2neo2}$ embryos. The imaging required removal of the thymic lobes

References

1. Anderson G, and Jenkinson EJ. Fetal thymus organ culture. *CSH Protoc.* 2007;2007(8):pdb prot4808.
2. Stuart T, Butler A, Hoffman P, Hafemeister C, Papalexi E, Mauck WM, 3rd, et al. Comprehensive Integration of Single-Cell Data. *Cell.* 2019;177(7):1888-902.e21.
3. Trapnell C, Cacchiarelli D, Grimsby J, Pokharel P, Li S, Morse M, et al. The dynamics and regulators of cell fate decisions are revealed by pseudotemporal ordering of single cells. *Nature Biotechnology.* 2014;32(4):381-6.
